# Satellite Gravimetry: A Review of Its Realization

**DOI:** 10.1007/s10712-021-09658-0

**Published:** 2021-10-07

**Authors:** Frank Flechtner, Christoph Reigber, Reiner Rummel, Georges Balmino

**Affiliations:** 1 Department 1: Geodesy, GFZ German Research Centre for Geosciences, 14473 Potsdam, Germany; 2 Institute of Geodesy and Geoinformation Science, Technical University Berlin, 10623 Berlin, Germany; 3 Institute of Geosciences, University Potsdam, 14469 Potsdam, Germany; 4 Astronomical and Physical Geodesy, Technical University Munich, 80290 München, Germany; 5 CNES-GRGS (Centre National d’Etudes Spatiales – Groupe de Recherches de Géodésie Spatiale), Toulouse, France; 6 OMP (Observatoire Midi-Pyrénées), Toulouse, France

**Keywords:** Gravitational field, Satellite gravimetry, Satellite altimetry, Gravitational field missions, CHAMP, GRACE, GOCE, GRACE FO, Satellite orbits, Satellite design, Mission objectives, Gravity field models, Mass changes, Satellite gradiometry, Laser interferometer

## Abstract

Since Kepler, Newton and Huygens in the seventeenth century, geodesy has been concerned with determining the figure, orientation and gravitational field of the Earth. With the beginning of the space age in 1957, a new branch of geodesy was created, satellite geodesy. Only with satellites did geodesy become truly global. Oceans were no longer obstacles and the Earth as a whole could be observed and measured in consistent series of measurements. Of particular interest is the determination of the spatial structures and finally the temporal changes of the Earth's gravitational field. The knowledge of the gravitational field represents the natural bridge to the study of the physics of the Earth's interior, the circulation of our oceans and, more recently, the climate. Today, key findings on climate change are derived from the temporal changes in the gravitational field: on ice mass loss in Greenland and Antarctica, sea level rise and generally on changes in the global water cycle. This has only become possible with dedicated gravity satellite missions opening a method known as satellite gravimetry. In the first forty years of space age, satellite gravimetry was based on the analysis of the orbital motion of satellites. Due to the uneven distribution of observatories over the globe, the initially inaccurate measuring methods and the inadequacies of the evaluation models, the reconstruction of global models of the Earth's gravitational field was a great challenge. The transition from passive satellites for gravity field determination to satellites equipped with special sensor technology, which was initiated in the last decade of the twentieth century, brought decisive progress. In the chronological sequence of the launch of such new satellites, the history, mission objectives and measuring principles of the missions CHAMP, GRACE and GOCE flown since 2000 are outlined and essential scientific results of the individual missions are highlighted. The special features of the GRACE Follow-On Mission, which was launched in 2018, and the plans for a next generation of gravity field missions are also discussed.

## The Pioneering Phase of Satellite Gravimetry

Today, Helmert's classical definition of geodesy (Helmert 1880) could perhaps be reformulated as follows: "Geodesy is the science of determining the geometric and gravimetric figure of the Earth and its orientation and how these properties change over time". The Earth's orientation corresponds to the determination of the axis of rotation with respect to an Earth-fixed coordinate system and a space-fixed coordinate system, which is referenced to a set of extragalactic radio sources. In view of the now achievable accuracies of geodetic measuring methods, the determination and analysis of the temporal changes of the geometric and gravimetric figure of the Earth and the Earth orientation are moving into the centre of geodetic research, in addition to the spatial variations. It is precisely these changes that have become increasingly important today—against the background of climate change and research into the drivers of the Earth system (Chao 2003). The entry into the space age was a quantum leap for geodesy. For the first time, it was possible to begin to actually measure the Earth globally and three-dimensionally and to consider the oceans as an equal target to the land surfaces. Please also refer to the historical overview "History of Earth Measurement" (Torge 2017).

In this paper we concentrate on satellite gravimetry, i.e. the determination of the Earth's gravitational field. A particularly fascinating special role is played by ocean altimetry, i.e. the centimetre-accurate scanning of the sea surface with satellites. Therefore, this method will be briefly characterized already here. On the one hand, altimetry provides us with highly accurate information about the geometric shape of the oceans and their changes in shape, and is therefore an important element for the determination of the global geometric Earth figure. On the other hand, the scanning of the ocean surface corresponds almost exactly to measuring the most important equipotential surface of the Earth's gravity field, the geoid, and thus to the determination of the gravimetric figure of the Earth. Only because of the relatively small sea topography, its magnitude is about ± 30 cm, the sea surface deviates from the geoid. If, for example, a model of the sea topography was available as an independent quantity, one could claim that satellite altimetry includes both the determination of the geometrical and the gravimetric shape of the oceans. Altimetry will be discussed again later. This introduction wants to give a short overview of the pioneering period of satellite gravimetry, from 1957 to the year 2000, the launch date of CHAMP, the first specialized gravity satellite mission. There are earlier reviews of satellite geodesy, or more specifically of satellite gravimetry. First of all, the freely accessible two-volume work (Henriksen 1977) is to be mentioned, a comprehensive presentation of the work of the first twenty years on satellite geodesy of all relevant US-American groups. The development of satellite geodesy from 1958 to 1982 is discussed in Lambeck and Coleman (1983) with replies of Lerch et al. (1986) and Lambeck and Coleman (1986), with a focus on the comparison and accuracy analysis of the first generation of gravity field models. An excellent review and outlook is given in Nerem et al. (1995). A very readable overview and outlook concentrated on the gravity field models is given in Rapp (1998). A European view on the development of satellite geodesy is presented in Barlier and Lefebvre (2001). Also the textbook (Seeber 1993) belongs to this series of general overviews including its comprehensive literature review.

### The Very First Developments

With the entry into the space age, Sputnik-1 was put into orbit by the Soviet Union on 4 October 1957—satellite gravimetry also began. By satellite gravimetry we understand the determination of spatial undulations and temporal changes of the gravitational field with the help of satellites. Already from the very sparse radio signals of Sputnik-1 and Sputnik-2 (launched on 3 November 1957) it was possible to determine the flattening of the Earth, much more accurately than from the preceding 150 years of classical geodetic triangulation networks on Earth (Buchar 1958; Merson and King-Hele 1958; Jeffreys 1959; King-Hele 1992). The following years were characterized by very rapid progress in the refinements of the analytical methods and the results obtained. The gravitational field is usually represented as a series of spherical harmonic functions, a double sum over the indices order $m$ and degree $n$ (Heiskanen and Moritz 1967, e.g. Equation (9–20) on p. 342). The series terms, which depend only on the latitude and thus on the degree *n*, correspond to Legendre polynomials with the zonal coefficients as weights. By far the largest zonal coefficient is the one of degree $n=2$ (order $m=0$), which represents the Earth’s flattening. The even degree series terms correspond to a representation of the gravitational field symmetrical to the equator. The weights depending on degree and order are called tesseral coefficients. Coefficients with degree equal to order ($n=m$) are called sectorial coefficients. Already in 1959, the pear shape of the Earth, resulting from the odd zonal coefficients, was proved. This was followed in quick succession by the determination of some zonal coefficients (Jacchia 1958; Cook 1958; O’Keefe et al. 1959a, 1959b). In 1961, a set of low degree and low order tesseral coefficients was determined for the first time (Kozai 1969; Izsak 1963; Guier and Newton 1965). An overview is given in King-Hele (1961). While the zonal terms in particular cause very long periodic perturbations in the satellite orbits, the tesseral terms cause short periodic effects. Due to the insufficient distribution of satellite observing sites over the globe and the limited accuracy of the measurements, it was initially difficult to detect short-periodic fluctuations in the satellite orbits at all. Therefore, the use of so-called orbit resonances had a special value. Orbit resonances occur when the satellite flies over the same geological structures in equal time intervals. More mathematically formulated, the average orbital velocity of the satellite should be commensurable with the rotation rate of the Earth. This permits an improved determination of certain linear combinations of gravitational field coefficients. Resonances were already used in the pioneering years for the determination of very low coefficients from orbital perturbations of geostationary satellites (Sehnal 1960; Groves 1960; Cook 1961). In the following years, the analysis of resonances was used as mathematical constraint to be met by certain coefficient groups and for the quality analysis of gravity field models (Anderle and Smith 1968; Balmino and Reigber 1975; Wagner and Klosko 1977; King-Hele et al. 1979).

A milestone in the development of satellite gravimetry was the Williamstown conference. At the invitation of NASA, a number of handpicked outstanding space experts, geodesists and Earth scientists met in Williamstown (Massachusetts) in 1969 to formulate a future program entitled "Solid Earth and Ocean Physics—Applications of Space and Astronomic Techniques" (Kaula 1969). The meeting was led by William Kaula. Several Europeans were also invited, for example Dan McKenzie from Cambridge, the Greek George Veis, the Italian Bepi Colombo, after whom the current planetary mission to Mercury is named, and the French scientist François Barlier. At that time, all major developments of the following decades in the field of satellite geodesy were already considered in a brilliant synthesis.

### Gravity Field Modelling in the 1970s to 1990s

**Fig. 1:**
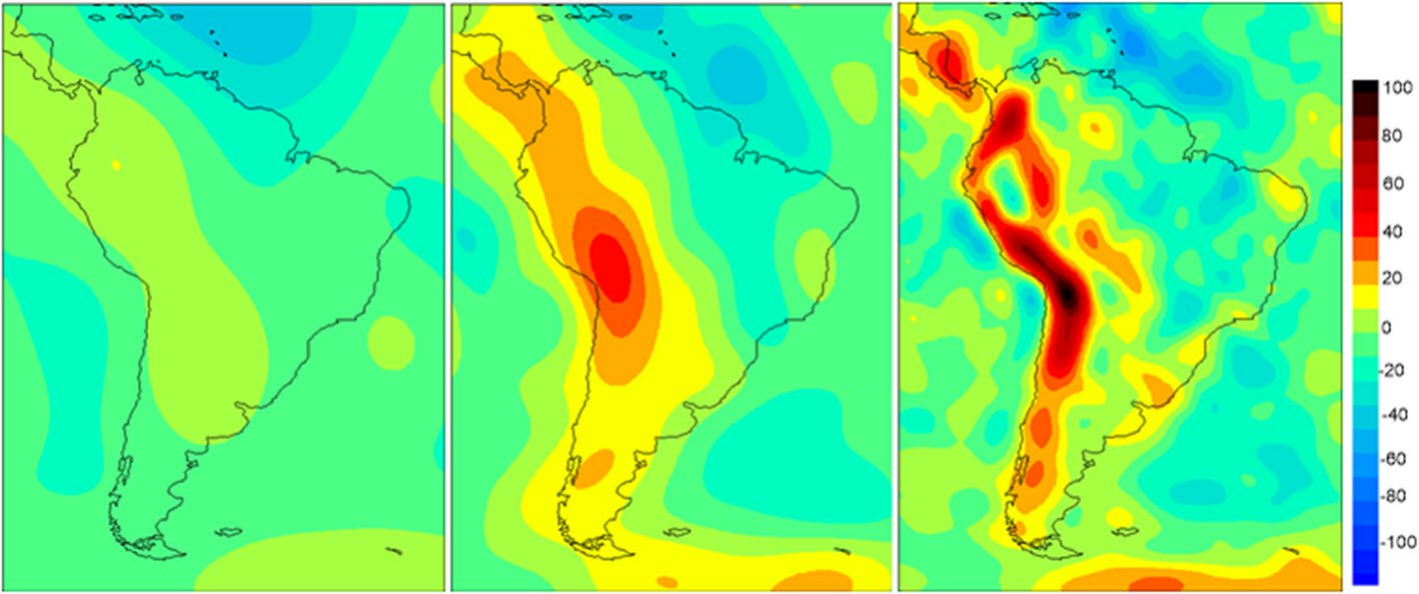
Geoid heights South America (in metre)—the development of the geoid models (here all shown up to degree and order $\mathrm{n}=\mathrm{m}=11$ of a spherical harmonic expansion): From the gravity field model SSE 1 to degree and order 15 (Lundquist and Veis 1966, left) via the Goddard model GEM 9 to $\mathrm{n}/\mathrm{m}=30$ (Lerch et al. 1979, centre) to the GRIM 5 s model with full resolution up to $\mathrm{n}/\mathrm{m}=32$ 32 and additional coefficients up to $\mathrm{n}=99$ and $\mathrm{n}=91$ (Biancale et al. 2000, right)

In the following years, more and more satellites became available for the reconstruction of the gravity field. In particular important for the evaluation was the addition of satellites whose orbital inclinations or orbital heights differed greatly. Of enormous value were, and still are the passive geodetic satellites Starlette (launch 1975), LAGEOS 1 (1976) and 2 (1992), AJISAI (1986), ETALON-1 and 2(1989), STELLA (1993), GFZ-1 (1995) and LARES (2012), which are solely equipped with laser reflectors. The two LAGEOS satellites are primarily used to maintain a stable geodetic reference system, to determine relativistic parameters and to record the secular change of the zonal coefficients of the gravity field (Yoder et al. 1983; Cazenave and Nerem 2002; Cox and Chao 2002; Dickey et al. 2002; Ciufolini et al. 2019). The addition of more satellites was one prerequisite, the development of new and very accurate observation techniques the other prerequisite for improving gravity field modelling. The radio signals of the first missions were replaced by directional measurements with cameras, microwave methods and laser distance measurements. After the U.S. Doppler system Transit, the German satellite central two-way distance and Doppler system PRARE, which flew on the altimeter missions ERS-1 and -2, and the French DORIS system (50 globally distributed stations with 2-frequency uplink) followed. Parallel to the new procedures, the global distribution of tracking stations improved, although there is still a strong concentration in the northern hemisphere. In the eighties, this development culminated in the establishment of the Global Positioning System GPS. GPS measurements on low-flying satellites made it possible for the first time to track their orbits practically uninterruptedly, three-dimensionally and very accurately. The first satellite equipped with a geodetic GPS receiver (ROGUE) was the altimeter satellite Topex/Poseidon (1992–2006), compare (Bertiger et al. 1994; Schutz et al. 1994). Table 1 summarizes the observation techniques. On the methodological level, the classical analytical procedures from celestial mechanics were very quickly replaced by very elegant analytical methods (Veis 1960; Kaula 1966, Lundquist and Veis 1966). This analytical approach (Lundquist and Veis ibid; Gaposchkin and Lambeck 1971) could generally be called the trademark of the gravity field modelling of the Smithsonian Astrophysical Observatory (short name of the models SSE = Smithsonian Standard Earth). A few years later the models of the Goddard Space Flight Center (GSFC, Greenbelt; short name of the models GEM = Goddard Earth Model, Lerch et al. 1972) and of the Center for Space Research (CSR, Austin; short name of the models TEG = Texas Earth Gravity models, Tapley et al. 1997a, b) followed, which were based on very efficient numerical methods. The joint gravity modelling effort of the GEM and TEG model development teams, resulting in the JGM-1&2 models, was done to support the precise orbit determination (POD) requirements for the TOPEX/Poseidon mission. In Europe, the models of the GRIM series (GRgs & Institute Munich = GRIM), also based on a numerical approach, resulted from a French–German cooperation (Balmino et al. 1976) A further logical, but in the implementation more complex step was the joint processing of satellite orbit perturbations and terrestrial gravity field quantities to a combined gravity field model. The problem was the patchy coverage of the Earth with terrestrial gravimetry and the accessibility, reliability and accuracy of these data. Only with the inclusion of altimetric geoid heights or gravity anomalies derived from altimetry, combined gravity field models with a high spherical harmonic expansion degree and improved accuracy were developed (Kaula 1967; Rapp 1979; Lerch et al. 1979; Rapp and Pavlis 1990; Wenzel 1999). This work has reached its peak with the models EGM96, EGM2008 (Lemoine et al. 1998; Pavlis et al. 2012) and EIGEN-6C (Förste et al. 2014). The three graphs in Fig. 1 of the geoid of South America show the rapid refinement of the gravity field models in the years before the CHAMP, GRACE and GOCE satellite missions.

**Table 1:** Observation methods of satellite geodesy (based on Nerem et al. 1995)

Procedure	Arrangement	Quality	Path accuracy	Advantage	Disadvantage
Camera	Satellite against star background	1–2”	1–2” ^(10 m)^	First exact Monitoring system	Refraction
SLR	Two-way distance	0.5 cm	2 cm	Very accurate	Depending on weather Station distribution
Radar S-Band	Two-way distance/change	1 m 0.3 cm/s	5 m 1 cm/s	Weather independent	Single Frequency System (ionosphere)
TRANET	Satellite to Ground Distance	0.2 cm/s	0.7 cm/s	Good Station distribution	Inaccurate Clocks Ionosphere
DORIS	One-way range (ground-to-satellite)	0.4 mm/s	0.5 mm/s	Good station distribution High accuracy Weather independent	Only one ground station at a time Only a few satellites
PRARE	Two-way range/range rate (Satellite—Ground—Satellite)	2–3 cm 0.1 mm/s	3–4 cm 0.2 mm/s	High accuracy Weather independent stations simultaneously Satellite central data provision	Only a few satellites Limited number of user stations
TDRSS	Two-/four-way range/range-rate	1 m (biased)	2 m 0.8 mm/s	Global coverage Good accuracy	Large orbit errors Weak link with reference frame
GNSS SST high-low	Pseudorange/phase (GNSS to Satellite)	1–2 cm	1–2 cm	High accuracy Continuous orbit tracking	Striping for low-low SST
Altimetry	Two-way range (Satellite to sea)	1–2 cm	2 cm	Precise scanning of Ice and oceans	Modelling near the coast
SST low-low	Pseudorange (Satellite to Satellite)	1 µm	1–2 cm	Excellent accuracy Time resolution	Striping
Gradiometry	Gravity Gradients	10 mE	1–2 cm	High spatial resolution	Very complex system

### The Satellite Altimetry Missions

The development of satellite altimetry and its scientific achievements are summarized in Fu and Cazenave (2001) and Stammer and Cazenave (2018). Table 2 gives an overview of the altimetry satellite missions. Since about 1991 (ERS-1 and T/P), radar altimetry has been used to scan the ocean surface with centimetre accuracy, either in geodetic mode with a very high spatial resolution but a rather modest repetition rate, or in oceanographic mode with a high temporal repetition rate but a relatively coarse grid of ground tracks. Several groups have since been calculating very accurate models of the marine gravity field (Sandwell et al. 2013; Andersen et al. 2010). Remaining uncertainties result from insufficient modelling of the sea topography, i.e. the height difference between sea surface and geoid. Oceanic geoid and gravity field models derived from altimetry are a very important data source for the calculation of combined gravity field models (Pavlis et al. ibid).

**Table 2:** Overview of the radar altimetry missions to date

Mission	Agency	Term	Characteristics
Skylab	NASA	1973	Proof of concept
Geos-3	NASA	1975–1979	1st altimetry mission
Seasat	NASA	1978	Short service life
Geosat	NASA	1985–1990	Main repeat 23 days
ERS-1	ESA	1991–1996	35 d
Topex/Poseidon	NASA/CNES	1992–2006	
ERS-2	ESA	1995–2011	10 d
GFO	USNavy/NASA	1998–2008	
Jason-1	CNES/NASA	2001–2013	10 d
Envisat	ESA	2002–2012	35 d
Jason-2/OSTM	CNES/Eumetsat/NASA	2008–	10 d
CryoSat-2	ESA	2010–	27 d
SARAL	CNES/ISRO	2013–	
Jason-3	CNES/Eumetsat/NASA	2016–	
Sentinel-3A	ESA	2016–	
Sentinel-3B	ESA	2018–	
Sentinel-6A	ESA	2020–	

## Gravitational Field Missions

### The Beginning of a New Epoch

At the beginning of the last decade of the twentieth century, the determination of the Earth's gravity field—based on classical directional, distance and Doppler observations of a large number of passive satellites in near-Earth space—had reached its natural limits. The reason for this was not the numerical and analytical orbital perturbation methods that had been used almost exclusively for the determination until then, but the incomplete and insufficiently accurate observation material, as well as the insufficient recording of the perturbation influence of the residual atmosphere on the satellite motion. The accuracy and resolution of the spatial structures and the expected temporal changes of the gravitational field lagged behind the results of the kinematics of the Earth's body.

This deficit and the necessary realization of specialized gravitational field missions with inter-satellite distance measurement and/or satellite gradiometry had been pointed out repeatedly in countless recommendations and studies since the early 1970s. Until the beginning of 1990, however, there was no chance of realizing one of the scientific-technical mission concepts first listed in the Williamstown Report (Kaula 1969) at NASA, ESA or any other space agency. Table 3 gives an overview of important workshops, studies and program steps.

**Table 3:** Development of satellite gravimetry—important workshops and program steps in the last century (see also Sneeuw and Ilk 1997)

Kaula W. (ed.): The Terrestrial Environment, Solid-Earth and Ocean Physics: Application of Space and Astronomic Techniques, Report of a Study at Williamstown, Mass., to the NASA, Cambridge, Mass., (1969) NASA: EOPAP: Earth and Ocean Physics Applications Program, Vol. II, Rationale and Program Plans, (1972) Lambeck K.: Solid Earth and Ocean Physics in the Post-Apollo Programme, ESRO/PA/R109, (1973) Abalakin V., G. Balmino, K. Lambeck, H. Moritz, J.D. Mulholland, F. Tozer: La Geodynamique Spatiale, Summer School Lecture Notes, Centre Nationale D'Etudes Spatiales, 20.8–13.9. 1974, Lannion, (1974) European Space Agency: SONG: Space Oceanography, Navigation and Geodynamics, ESA SP-137 (European Workshop, Schloss Ellmau, (1978) National Research Council, Committee on Geodesy: Applications of a Dedicated Gravitational Satellite Mission, National Academy Press, Washington D.C., 53 pp., (1979) National Research Council: A strategy for Earth science from Space in the 1980's, part I: Solid Earth and oceans, National Academy Press, Washington D.C., 99 pp., (1982) Wells W.C. (eds.): Spaceborne Gravity Gradiometers, NASA Conference Publication 2305, (Proceedings of a Workshop sponsored by the OSSA Geodynamics Branch, NASA-GSFC, 28–2—2–3, (1984) NASA: Geopotential Research Mission (GRM), NASA Conference Publication 2390, (Conference at the University of Maryland, October 29–31, (1984). European Space Agency: SESAME: Solid Earth Science & Application Mission for Europe, ESA SP-1080 (ESA Special Workshop, Ising am Chiemsee, (1986) NASA: Geophysical and Geodetic Requirements for Global Gravity Field Measurements 1987–2000, (Gravity Workshop, NASA-GSFC, Colorado Springs, (1987) CIGAR I: Study on precise gravity field determination methods and mission requirements, Final report, ESA Contract No. 7251/87/F/FL, (1989) ARISTOTELES: Proceedings of the Italian Workshop on the European Solid-Earth Mission ARISTOTELES, AERITALIA, Trevi, (1989) Anderson A.J., R. Sabadini, S Tinti, S. Zerbini, J. Achache, A. Geiger, F. Arnet, E. Klingele: Study of the geophysical impact of high-resolution Earth potential fields information. ESA study, (1990) CIGAR II: Study on precise gravity field determination methods and mission requirements, Phase 2—final report, ESA Contract No. 8153/88/F/FL, (1990) Lambeck K.: Aristoteles: An ESA Mission to Study the Earth's Gravity Field, ESA Journal 14:1–21, (1990) NASA: Coolfont 1989 Workshop Outcome: Solid Earth Science in the 1990s, NASA TM 4256, Program Plan, (1991) European Space Agency: The Solid-Earth Mission ARISTOTELES, ESA SP-329, International Workshop, Anacapri, (1991) CIGAR III: Study of the gravity field determination using gradiometry and GPS, Phase 1/2—final report, ESA Contract No. 10713/93/F/FL, (1993) Frey H., J. Abshire, B. Bills, J. Connerney, B. Johnson, R. Langel, F. Lerch, S. Nerem, E. Pavlis, D. Skillman, D. Smith, P. Taylor, and C. Voorhies: Mission proposal GAMES (1993) Rummel R., P. Schwintzer (eds.): A Major STEP for Geodesy, report of the STEP Geodesy Working Group, pp. 54—54, (1994) CIGAR IV: Study of advanced reduction methods for spaceborne gravimetry data, and of data combination with geophysical parameter, Final report, ESA Contract No. 152163, (1996) Bills B. G. and H. J. Paik: Mission proposal GEOID (1996) National Research Council, Committee on Earth Gravity from Space: Satellite Gravity and the Geosphere, Contributions to the Study of the Solid Earth and Its Fluid Envelope, National Academy Press, (1997) Colombo O. L. and B. F. Chao: Mission proposal TIDES (1997) European Space Agency: Reports for mission selection—The Four Candidate Earth Explorer Core Missions, ESA SP-1233(1), ESA Publication Division, Noordwijk, (1999)	

How should such concepts look like? Probably the most important features of these envisaged concepts were:

- the choice of an orbit altitude as close to Earth as possible (altitudes between 250 and 500 km) in order to minimize its attenuation of the Earth’s gravity field at satellite altitude
- an almost global and even coverage of the Earth with ground tracks by using a polar or near-polar inclination of the orbital plane and an almost circular orbit,
- an uninterrupted, three-dimensional and global determination of the position of the near-Earth satellite by means of orbit tracking from the high orbiting system of navigation satellites (high-low concept)
- the continuous recording of the non-gravitational forces acting on the satellites with precision accelerometers, and
- the additional amplification of the gravitational field signal either by a differential distance measurement between satellites in low Earth orbit (low-low concept) or the in-situ measurement of gravitational gradients using a gravitational gradiometer, again at low orbit altitude.

It was not until 1992 that NASA initiated the era of high-low satellite observation with GPS satellite signals received with the first space-borne GPS receiver of the ROGUE class from the Jet Propulsion Laboratory (JPL) on board the TOPEX/POSEIDON (Yunck et al. 1994) altimeter satellite. The time of more precise GPS orbital products did not begin until the mid-1990s with the establishment of the civil GPS service IGS of the International Association of Geodesy (IAG), see Neilan et al. (2000). However, this successful first flight of a dual-frequency GPS receiver on TOPEX/POSEIDON marked the beginning of a new era of satellite orbit determination of low-flying satellites, which was to become a trendsetter for the success of the new generation of gravity field missions described below. The first flight of the French GRADIO/ASTRE accelerometer on the STS78 shuttle mission in June 1996 (Touboul et al. 1996) was of similar significance for further development. With the possibility of a very accurate high-low orbit tracking by GPS and the successful realization of space-qualified, very accurate accelerometers, the decisive building blocks for the realization of specialized gravitational field missions were available at the end of the nineties. With the CHAMP, GRACE and GOCE missions they became reality.

### The CHAMP Mission

#### A Product of German Reunification Activities

Observation and modelling of the Earth's global gravitational and magnetic field played an important role in the future program of the German Research Center for Geosciences (GFZ = Deutsches GeoForschungsZentrum Potsdam), a large-scale research facility established in the course of Germany’s reunification in 1992. After the German Agency for Space Affairs (DARA)—later integrated into the German Aerospace Center (DLR = Deutsches Zentrum für Luft- und Raumfahrt)—had taken the initiative at the beginning of 1994 to finance a lead project for the space industry in the former German Democratic Republic (GDR) states, the GFZ had the unique opportunity to propose a geopotential mission with state-of-the-art observation technology in the planned competitive procedure. Together with the DLR and a consortium of originally 12 industrial companies from the former GDR states, the feasibility of such a mission was investigated in 1994. In 1995/1996 DARA selected it as a small satellite mission to be realized under the leadership of the GFZ. Specifications of the small satellite approach were: fast realization, low costs, ambitious mission objectives. Due to the unique combination of novel instruments for the simultaneous detection of gravity and magnetic field, as well as for the sounding of the atmosphere and ionosphere, the mission was named CHAMP (**CHA**llenging **M**inisatellite **P**ayload). Launched in the year 2000, CHAMP started the Decade of Geopotentials, proclaimed by the IUGG in 1999, on schedule and provided Earth System Research with a unique and continuous geophysical data set over the entire decade.

#### Mission Objectives, Satellite Design and Measurement Principle

The primary mission goal of the CHAMP mission was a significantly improved determination of the long-wave components (> 800 km) of both the Earth's gravity and geomagnetic field using innovative instrumentation on board the satellite. Secondary mission goal was the use of the on-board GPS instrumentation for the first operational use of radio occultation technology for remote sensing of the atmosphere and ionosphere.

**Fig. 2:**
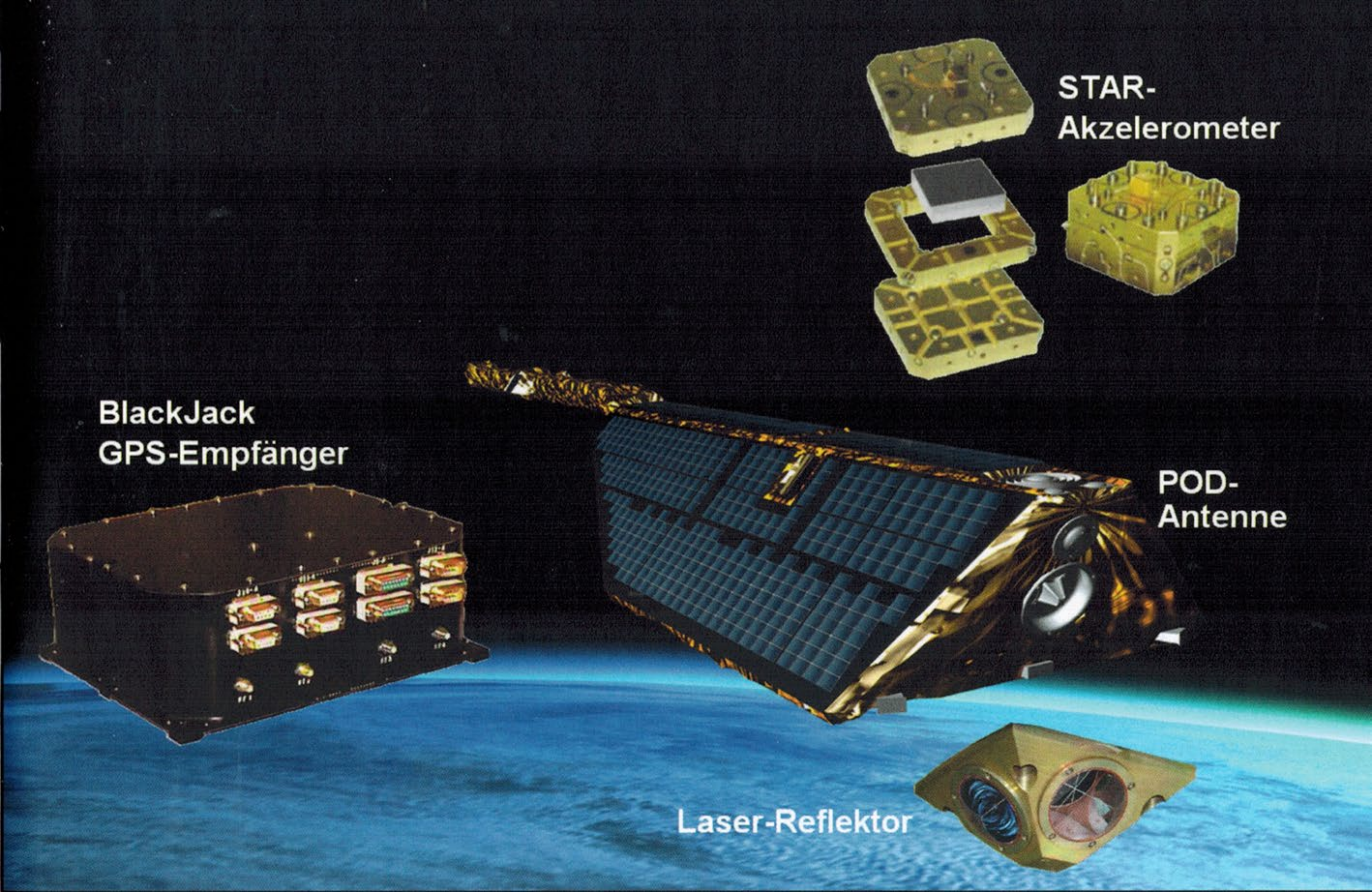
CHAMP gravity field measurement systems

In order to take into account the conditions of the specified small satellite approach, the project structure, depth of documentation and the work locations corresponding to the project schedule were selected in such a way that a time- and cost-efficient course of the project work was possible. The system design work was carried out in Potsdam by GFZ and industrial team (IT) employees, with input from the component and instrument manufacturers and DLR. This cooperation at one work site enabled an unbureaucratic and fast exchange of information, high flexibility and short reaction times for all decisions concerning satellite design. During the development of the CHAMP mission, attention had to be paid to an optimal adaptation of the satellite design to the two primary mission objectives, simultaneous measurement of gravity and magnetic field, and the secondary mission objective, sounding of the upper atmosphere. Constructive drivers in this context were a well-defined and constant centre of gravity position, a three-axis stabilized attitude control with only negligible lateral accelerations, a long boom for magnetically clean measurements and aerodynamic conditions that should ensure a long mission duration at low orbit altitude. In order to optimize the aerodynamic behaviour and the magnetic field observation environment, the satellite was built as a relatively heavy trapezoidal body, measuring 430 cm × 75 cm × 162 cm (l/h/w), with a 404 cm long fold-out boom in flight direction (see Fig. 2). The satellite weighed, including two tanks with 34 kg cold gas for attitude control and orbital manoeuvres, 522 kg at the beginning of the mission. The average power consumption of about 120 W was supplied by 7 square-metre solar cells and a 16 Ah NiH2 battery.

The Earth-oriented alignment of the satellite was ensured by three magnetic torquers and 12 cold gas control nozzles. The orientation of the satellite in space was determined by star sensors on the satellite body and on the boom. These star sensors were manufactured and calibrated by the Danish Technical University (DTU).

In order to be able to stay within the given budget of the small satellite project, competent and interested partners for the provision of equipment were sought and found. The exact position, velocity and a uniform time reference for all devices on board was provided by a two-frequency GPS receiver of the newly developed BlackJack series, which was provided by the NASA Jet Propulsion Laboratory (JPL). The electrostatic accelerometer STAR, manufactured by the French company ONERA and provided by the Centre National d'Etudes Spatial (CNES), had its maiden flight on CHAMP. It fulfilled the specified resolution of < 3 × 10^–9^ $\mathrm{m}/{\mathrm{s}}^{2}$ for the two highly sensitive axes (Förste et al. 2005). From autumn 2000 it provided valuable information on the accelerations of the non-gravitational surface forces, information which is of great importance for the accurate recovery of the gravitational field and the development of air density models. The ion drift meter DIDM and a Langmuir probe, developed and provided by the US Air Force Research Laboratory (AFRL), as well as the fluxgate and Overhauser magnetometers on the boom (manufactured and provided, respectively, by DUT and the French LETI—Laboratoire d'Electronique et de Technologie de l'Information), were the main instruments for the electrical and magnetic measurements on CHAMP. A laser retro-reflector manufactured by GFZ on the bottom-side of the satellite which supports the SLR (Satellite Laser Ranging) measurement principle completed the instrumentation. A highly autonomous control and data processing system guaranteed safe operation over long periods of time (up to 12 h) without contact to ground stations. The data was stored in a mass memory with a capacity of 1.2 Gigabit and sent to the receiving stations in Weilheim, Neustrelitz and Ny Ålesund during overflights.

The pre-integration of the mechanical CHAMP structure was carried out at the Dornier company in Friedrichshafen (today Airbus Defense & Space GmbH), and that of the cold gas system at the space company RST in Rostock. The electrical integration and system tests were carried out in Jena at the company Jena Optronik, and the environmental tests were finally performed at the Industrieanlagen-Betriebsgesellschaft (IABG) in Ottobrunn. After three and a half years of construction and testing, CHAMP was ready for shipment to the launch site in May 2000.

#### Launch, Mission History and Data Provision

**Fig. 3:**
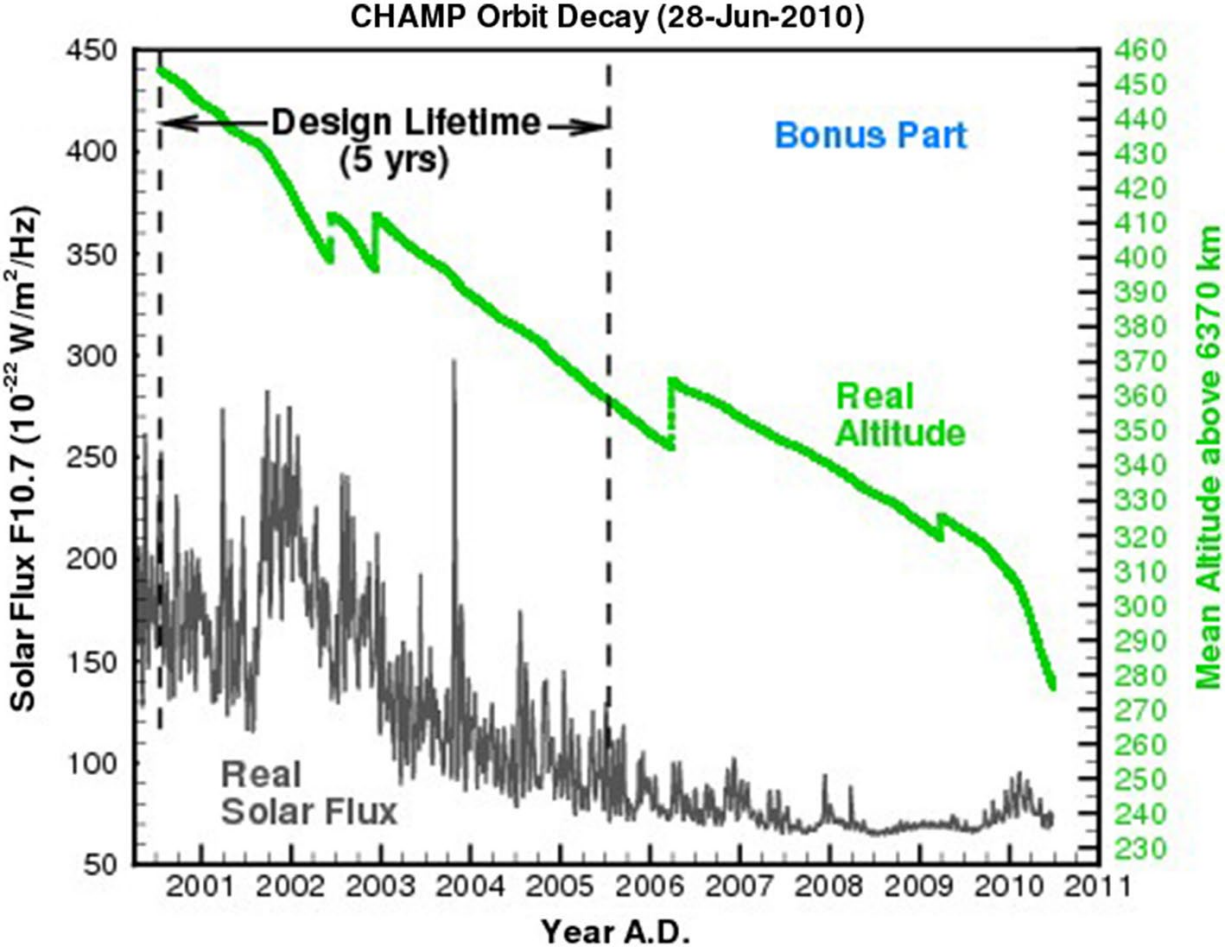
CHAMP orbit altitude changes 7/2000–7/2010

COSMOS International, a joint venture between OHB Systems GmbH and the Russian POLYOT Production Cooperation, was responsible for supplying the rocket and preparations on the launch site. Following intensive preparations on the Plesetsk Cosmodrome, the largest central Russian rocket launch site 800 km north of Moscow, CHAMP was launched on 15 July 2000 on a COSMOS rocket into its polar (orbit inclination $i=87.3^\circ$) and almost circular (orbit eccentricity$e=0.004$) orbit at an altitude of 454 km. The sharp increase in solar activity between mid-2001 and the end of 2002 and the resulting increased deceleration of the satellite made several orbital manoeuvres necessary. With a sequence of jet firings in the apogee, the CHAMP orbit was raised by about 13 and 16 km in June and December 2002, respectively. These corrections transformed the initially slightly elliptical CHAMP orbit into an almost perfect circular orbit *(*$e=0.0$) which is the so-called frozen orbit, i.e. the orbit with smallest possible eccentricity. To maximize the mission duration two more orbital manoeuvre were performed in March 2006 and March 2009 (see Fig. 3). The most important characteristics of CHAMP are summarized in Table 4.

**Table 4:** Characteristics of CHAMP

CHAMP	CHAllenging Minisatellite Mission
Main instrument for gravity field recovery	Low Earth orbiting satellite with onboard geodetic GPS receiver for high–low SST
Other instruments (for magnetic field recovery)	Digital ion drift meter, overhauser magnetometer, fluxgate magnetometer
Orbit determination	Spaceborne geodetic GPS receiver
Orbit control	Laser retro reflector
Orientation in space	Four stellar sensor systems
Measurement of non-gravitational accelerations	Electrostatic STAR accelerometer
Mission duration	15.7.2000–19.9.2010
Orbit height	Descending from an altitude of 454 km after launch to 260 km in July 2010, after raising the orbit height twice in July and December 2002 and in addition once in July 2006 and July 2009 in order to maximize mission duration
Orbit eccentricity	Quasi-circular to frozen orbit

CHAMP had reached a flight altitude of 260 km on the 10th anniversary of its launch. On 19 September 2010, it plunged into the Earth's atmosphere and burned up.

A successful satellite mission requires not only a perfectly functioning satellite in space but also a complex, well-functioning infrastructure on Earth. This so-called CHAMP ground segment was designed from the very beginning in such a way that, in addition to the mission control data, all sensor data could be made available to the scientific users as quickly as possible in different processing stages over a long mission period. This ground infrastructure consisted of components which, on the one hand, ensured the operational control of the proper functioning of CHAMP and the flow of data from the satellite to ground stations. In addition, there were components that ensured the processing of the satellite sensor data into scientific data products and their archiving and distribution to the users. The German Space Operations Center (GSOC) of DLR in Oberpfaffenhofen was responsible for the work of the MOS mission operating system. A special scientific data system was developed by the GFZ and operated continuously and largely automatically during the mission. With this system the raw sensor data sent by CHAMP were decoded in the data operating system SOS and converted into calibrated physical measurement data together with data from the laser and GPS ground station networks. These formed the basis for the derivation of the scientific standard products of different processing stages for the gravitational field, magnetic field and atmosphere in the processing system SDS. An ISDC information and data management system specially developed for the CHAMP mission ensured the archiving/administration and efficient provision of the measurement data and data products for all users.

#### Special Features of the CHAMP Mission for Gravity Field Modelling

CHAMP was equipped with a total of seven different scientific instruments, whose data were processed in operational mode from May 2001 onwards and evaluated by groups of scientists worldwide (Reigber et al. 2001). First results of these groups on the modelling of gravity and magnetic fields as well as on atmosphere/ionosphere soundings have been summarized in several conference proceedings (Reigber et al. 2005; Flury et al. 2006). Here the importance of the CHAMP mission for gravity field determination will be briefly discussed.

First of all, we would like to emphasize a few features that clearly set the CHAMP mission apart from all previous missions and made it the decisive precursor for subsequent satellite-to-satellite tracking (SST) missions.

**Fig. 4:**
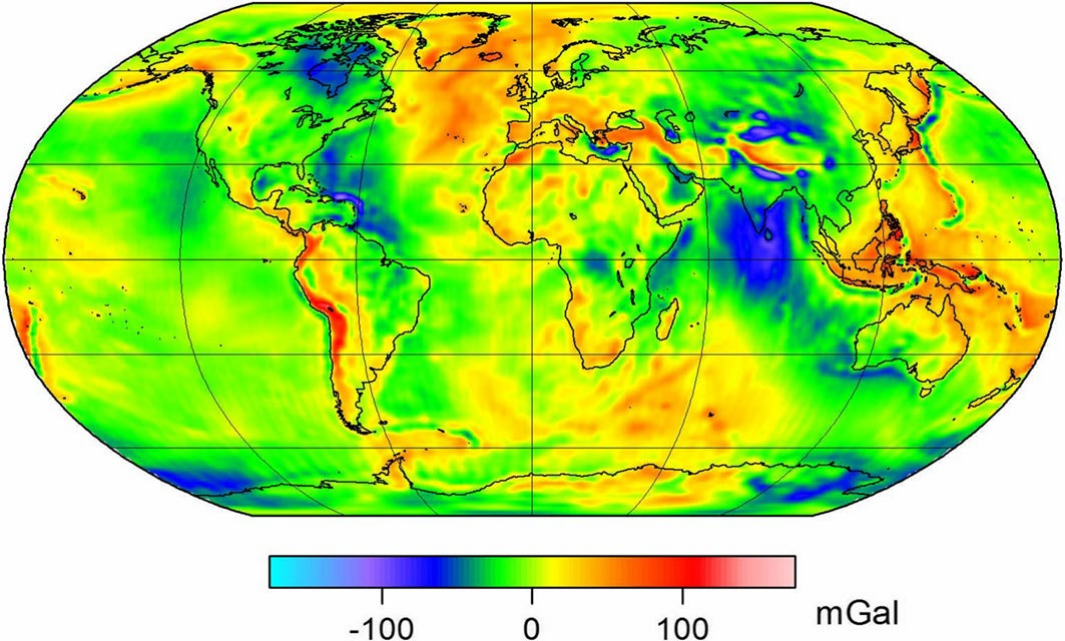
Gravity field model from 860 days of CHAMP data

- It was the first time that operational and scientific data was collected on board of a geoscientific long-term mission in low orbit, almost continuously (approx. 98 per cent) every second and that all measured quantities were provided with a uniform and accurate (< 1 ms) time stamp.
- This information was fed into a network of ground system components, realized for the first time in Germany for a geoscientific mission, for the ongoing control of satellite functions and the ongoing monitoring, processing and provision of instrument data and scientific reference products to interested research groups. The various components were developed and operated throughout the entire mission period by (1) the DLR/ GSOC in Oberpfaffenhofen for the satellite operation, (2) the GFZ Potsdam for the scientific data processing, archiving and distribution system, and (3) the DLR/DFD branch office Neustrelitz for raw data archiving and processing.
- In addition to the DLR Receiving and Commanding Station Weilheim and the DLR/DFD Receiving Station Neustrelitz, the development and remote operation of a data receiving station on Spitsbergen and a globally distributed network of near-real-time GPS ground stations was promoted by the GFZ for the CHAMP mission.
- The multi-year continuous data sequence of GPS BlackJack on-board receiver and STAR accelerometer at one-second intervals provided by the CHAMP mission has enabled the continuous determination of exact kinematic satellite positions and the exposure of the purely gravitational signal in the satellite orbit, and thus for the first time the application of so-called two-step or in-situ procedures.
- As Fig. 4 demonstrates, just a few months of continuous high-low-tracking of CHAMP yielded a global gravity field that was superior to the cumulative effort of the 4 decades before.

This was the starting point for a large number of new evaluation groups at universities and research institutions in Germany and abroad to establish themselves alongside the operational CHAMP evaluation team at the GFZ, and to implement the latest methods of orbit and field parameter determination within the framework of special utilization programs. Already the first months of operation of the CHAMP satellite confirmed in an impressive way that, as planned, accurate GPS-CHAMP inter-satellite measurements and STAR accelerometer measurements could be obtained almost continuously from low, near-polar orbit. Already from these first monthly data, a gravity field based on data from a single satellite could be calculated for the first time, which showed an improvement in the long wavelength proportions by a factor of 10 compared to pre-CHAMP models (Reigber et al. 2002). Several years of CHAMP data series, processed by the classical method of differential orbit and field parameter correction, provided further improved models of the static gravity field up to spatial resolutions of about 500 km (Flechtner et al. 2010).

The CHAMP mission opened up the possibility of calculating exact 3D satellite positions along the orbit, and this was quickly reflected in a broad implementation of in-situ methods that had previously not been used. The results obtained with these methods were to a large extent comparable to the results obtained with higher computational effort using the classical numerical orbit perturbation method. In particular, the solutions based on the energy integral method (Gerlach et al. 2003; Földvary et al. 2005), the acceleration method (Reubelt et al. 2003) and the generalized Fourier analysis of short CHAMP orbits (Ilk et al. 2005) are worth mentioning. In particular, with the latter method of field parameter determination based on the orbit determination as boundary value task, very good results could be obtained both for the global gravity field and for regional partial solutions (Mayer-Gürr et al. 2005).

With the results already obtained in the first year for the Earth's potential fields (Fig. 4) the importance of CHAMP as a pilot mission for a number of successor satellites in preparation became clear. The NASA/DLR mission GRACE (launch 2002) and ESA mission SWARM (launch 2011) are visible examples of this, but also—from the point of view of orbit and baseline determination—the DLR remote sensing missions TerraSAR-X (launch 2007) and Tandem-X (launch 2010).

### The GRACE Tandem Mission: How It Came About

With the CHAMP mission, launched in 2000, for the first time a low Earth orbiting satellite equipped with a precision accelerometer had been continuously tracked simultaneously by up to ten GPS satellites in high orbit. It was a breakthrough in the determination of the large-scale structures of the Earth's static gravity field using a wide variety of evaluation methods (Löcher 2010).

As early as the late 1960s, a publication by Wolff (1969) and the landmark Williamstown conference (Kaula 1969), mentioned at the beginning of this article, had pointed out that the inter-satellite ranging signal between a pair of satellites orbiting the Earth in the same orbital plane contains significant information about the medium to short wavelength components of the Earth's gravitational field. This mission concept was adopted by US scientists for the early GRAVSAT proposal (Fischell and Pisacane 1978) and that of the SLALOM mission in Europe (Reigber 1978). However, these two proposed experiments and the subsequent considerations for the US Geopotential Research Mission GRM (Keating et al. 1986), the NASA/GSFC laser SST concept GAMES (Frey et al. 1993) and the European mission studies for POPSAT (Reigber et al.^1987^), BRIDGE (Balmino et al. 1995) and ARISTOTELES (European Space Agency 1991a, b) could not be placed successfully in any of the ESA or NASA funding programs.

On the occasion of the IUGG meeting in Boulder in August 1995 a presentation of the GFZ- work on the CHAMP project and the considerations at JPL on the development of an intersatellite ranging instrument was given. Following these presentations, it was agreed between GFZ, JPL, and CSR that a GFZ-funded feasibility study should be conducted to investigate different variants of a Tandem SST (Satellite-to-Satellite) mission based on the JPL Ranging Instrument and the technology that was under development for the CHAMP project. This feasibility study was completed in February 1997 with participation of GFZ, JPL, CSR, the industrial team involved in the CHAMP development and the DLR’s GSOC. It provided the technical and programmatic details for the project, which was agreed upon by CSR, GFZ Potsdam, JPL and Space Systems/Loral and submitted to NASA within the framework of the Earth System Science Pathfinder Program (ESSP). It was a proposal for an American-German partnership mission with the acronym GRACE (**G**ravity **R**ecovery **A**nd **C**limate **E**xperiment) (Tapley et al. 1997a,b). The breakthrough for the realization of this first low-low SST mission came in spring 1997 with the acceptance of the GRACE proposal by NASA as the first mission of its ESSP program, and after the signing of the Memorandum of Understanding between the space agencies, NASA and DLR at the end of 1998.

CSR had overall responsibility for the GRACE mission, which was finally launched in March 2002 and operated very successfully until fall 2017. JPL was responsible for the US-American parts of the project (satellites and instrumentation) and the GFZ was responsible for the German mission elements (satellite launch and mission operation). Data processing, distribution, archiving and product verification were carried out in cooperation between CSR, JPL and GFZ.

#### GRACE: Mission Objectives, Satellites and Measurement Principle

GRACE was a satellite mission specifically designed to measure the temporal variations of the Earth's gravitational field. The main scientific goal was to monitor globally integrated mass changes in the geosphere, which are associated with climate-relevant processes, over a measurement period of several years. The primary metrological objective was to map the global gravitational field with hitherto unprecedented accuracy over a spatial range of 400 to 40,000 km every 30 days. The overall system consisting of two identical satellites and the on-board instruments was therefore designed to obtain monthly mean images of the gravitational field, the accuracy of which in this wavelength range should exceed the knowledge gained from the CHAMP mission by a factor of 100 to 1000. In addition to the determination of averaged (static) gravity field models from the joint processing of monthly data series over the entire mission period, the secondary mission objective, as with CHAMP, was the use of GPS radio occultation measurements to obtain density and temperature profiles in the upper atmosphere.

Month by month a new global image of the Earth's gravity field, which changes both spatially and temporally, should be created from GRACE data. During the planned five-year mission lifetime, the sequence of these highly detailed maps of the gravitational field was to be used to detect minute gravitational fluctuations and relate them to the physical properties of planet Earth, following the considerations and simulations made in the NRC report (National Research Council 1997). As impressively explained in this report, but also already thought of by Helmert (1910), these temporal changes occur as a consequence of the short- and long-term mass exchange between geosphere, hydrosphere and atmosphere. The tiny changes in the global gravitational field (in the form of temporal changes of the geoid or gravity anomaly field) represent such mass shifts. They can thus be used, for example, for investigations of changes in the mass balance of the hydrosphere, the oceans, the cryosphere and of changes in the heat and mass exchange between the ocean and the atmosphere.

**Fig. 5:**
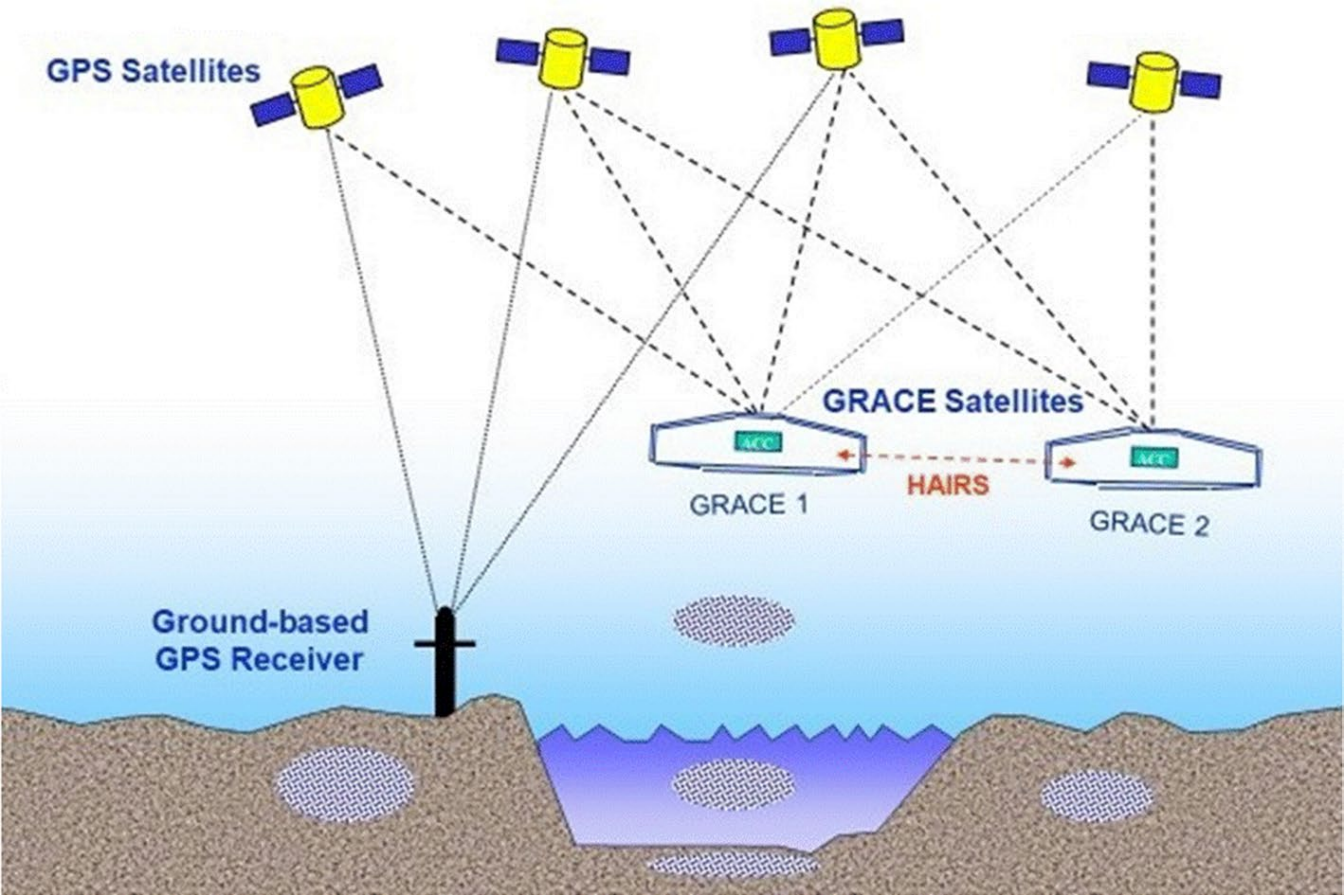
GRACE mission concept (HAIRS = high accuracy inter-satellite ranging system)

**Fig. 6:**
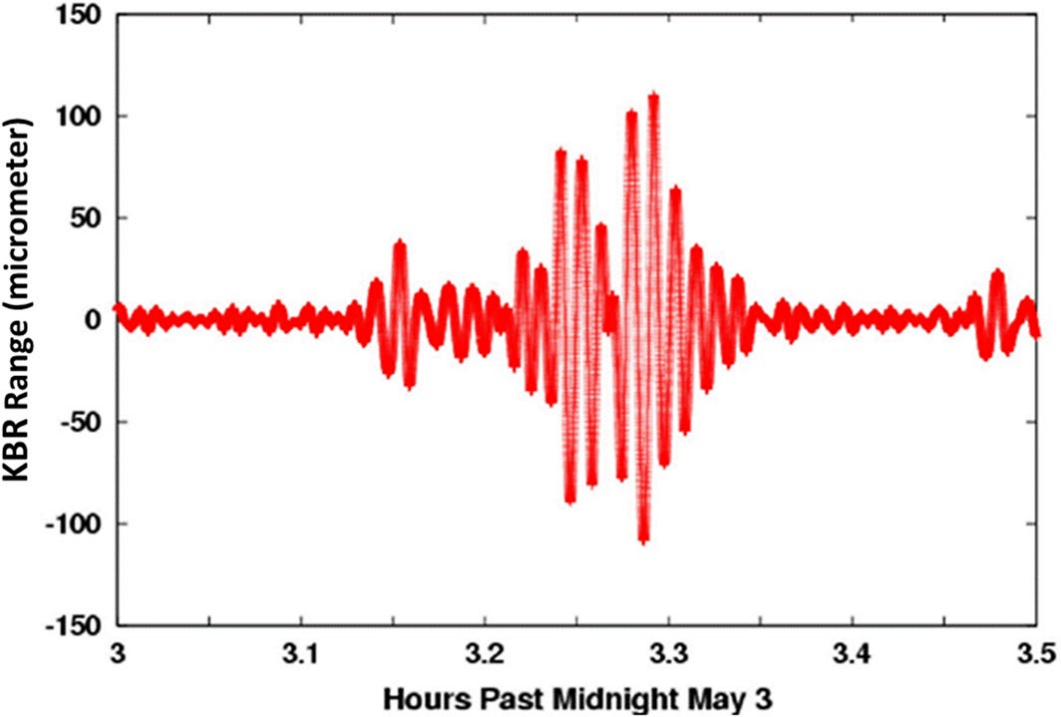
Distance change in micro-metre between the satellites during an overflight over the Himalayas on 3 May 2003

The GRACE mission consisted of two identical satellites orbiting the Earth at a distance of about 220 km in the same orbit, initially at an altitude of about 500 km, and connected by a high-precision microwave ranging system. Figures 5 and 6 show the GRACE measurement concept. When approaching a positive mass anomaly on or within the Earth, the nearer satellite is accelerated more strongly by the gravitational force than the twin following it. If the first satellite crosses the mass anomaly, it begins the deceleration phase, while the second is accelerated. If both satellites move away from the mass anomaly, the second satellite is initially decelerated even more than the first satellite, which is now further away. This leads to the signature in the measured distance change of both satellites shown in the Fig. 6, after subtracting the main signal due to the Earth’s flattening in the order of 2 km. Because of the differential character of the measurement, much finer structures can be resolved with the twin configuration than from the orbital disturbances of a single satellite. However, measurement accuracies in the order of a few micrometres are required.

**Fig. 7:**
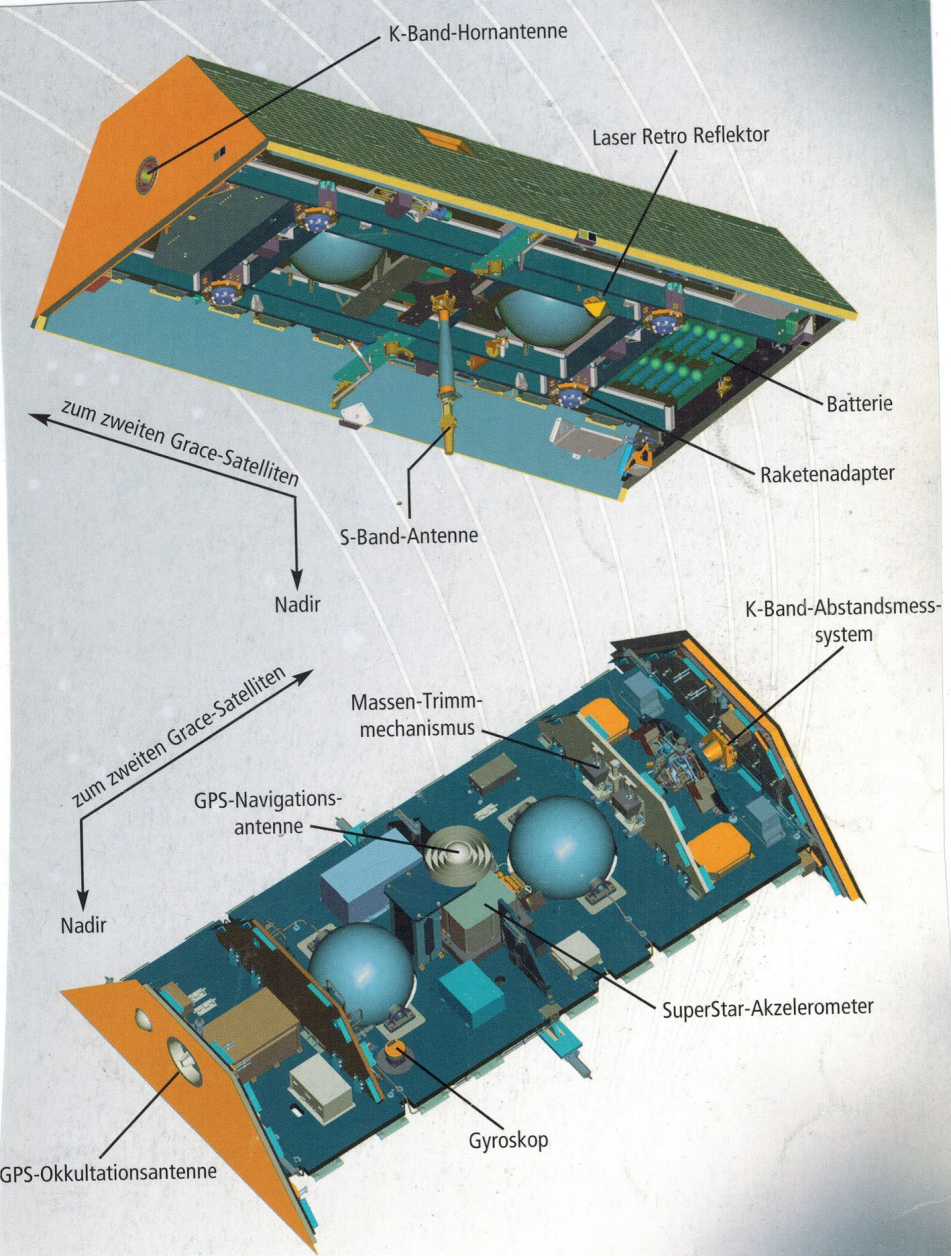
Structure of the GRACE satellites ( *source*: Astrium)

Each of the two completely identical satellites, built by Astrium (now Airbus Defense & Space GmbH), on behalf of Space Systems Loral, was 3.1 by 1.9 m in size and weighed 480 kg at launch, including 32 kg of fuel (Fig. 7). The structure of the GRACE satellites was made of carbon fibre-reinforced plastic (CFRP), a material that allows the creation of highly rigid structures at low weight.

This thermostable stiffness of the structures (twisting < 3 µm per revolution) was a prerequisite for the realization of the precise fine alignment of the satellites to each other, and the continuous acquisition of highly accurate measurements of relative distance and speed between the two satellites in the range of a few micrometres and 0.1 µm/s. In addition, a special mass trim mechanism ensured that the centre of gravity of each satellite did not shift by more than 0.1 mm during the entire mission. The main measurements were made with the JPL-built High Accuracy Inter-Satellite Ranging System (HAIRS), which measures the change in distance between the two satellites. Two different frequency signals of 24 GHz (K-band) and 32 GHz (Ka-band) were transmitted and received between the satellites (Davis et al. 1999). The received and initially stored signals were regularly transmitted to the ground and then combined to range measurements, unaffected by ionospheric effects. They formed the basis for the gravity field measurements. However, the orbit of both satellites does not only depend on the globally integrated gravitational signal of the mass distribution and mass motions in the Earth system, but also on accelerations caused by the air drag of the high atmosphere and the radiation pressures of the Sun and of the Earth. These latter accelerations have to be measured very accurately to separate the gravitational effects from the non-gravitational effects in the distance changes. For this purpose, a high-precision SuperSTAR accelerometer from the French company ONERA (Touboul et al. 1999a,b), a further development of CHAMP’s STAR accelerometer, was mounted in the centre of mass of each satellite. American BlackJack GPS receivers provided the orbit positioning of both GRACE satellites, as with CHAMP, with an accuracy of a few centimetres. The orientation of each GRACE satellite was recorded with the help of two Danish star cameras from DTU. They were rigidly attached to the accelerometer and observed the sky on the port side and on the starboard side at an angle of 55° to the zenith. Finally, a GFZ manufactured laser retroreflector was attached to the bottom side of the spacecraft to allow independent verification of the GPS-determined GRACE orbits using the terrestrial laser tracking network data. With this set of continuous measurements of the GRACE tandem it was possible to achieve globally largely homogeneous distributions of highly accurate measurement data month by month, which formed the basis for the calculation of monthly, up to daily models of the global gravitational field (Tapley et al. 2004).

#### Launch, Mission History and Data Products

The two GRACE satellites were launched on 17 March 2002 from the Plesetsk Cosmodrome in northern Russia with a ROCKOT launch vehicle, a converted Russian intercontinental rocket SS-19 with a manoeuvrable BREEZE-KM upper stage. The German-Russian space company EUROCKOT Launch Services, a joint venture of Astrium and Khrunichev, was responsible for the provision of the rocket and the preparatory work at the launch site. The satellites were injected with pinpoint accuracy into a very close polar ($i=89^\circ$) and almost circular ($e=0.0003$) orbit at an altitude of 500 km. From this point on the satellites were taken over by DLR’s GSOC in Oberpfaffenhofen for further mission operations. After alignment of the satellites along their local vertical and horizontal axes, and separation to a mutual distance of 220 km, both satellites drifted freely under the influence of the gravitational field during the entire mission period. The attitude control systems of both satellites, consisting of magnetic torquers and cold gas nozzles, continuously controlled the attitude so that the mutual alignment of the horn antennas of the HAIRS system remained within a range of 1 to 10 mrad. In order to keep the slowly varying distance between the satellites in the range of 220 ± 50 km during the entire mission period, the satellites were repositioned to nominal distance about twenty times. In order to avoid the risk of losing thermal control of the horn antennas due to the effect of atomic oxygen, the mutual position of the satellites was exchanged in December 2005, and additionally four times towards the end of the mission, by a special manoeuvre.

**Fig. 8:**
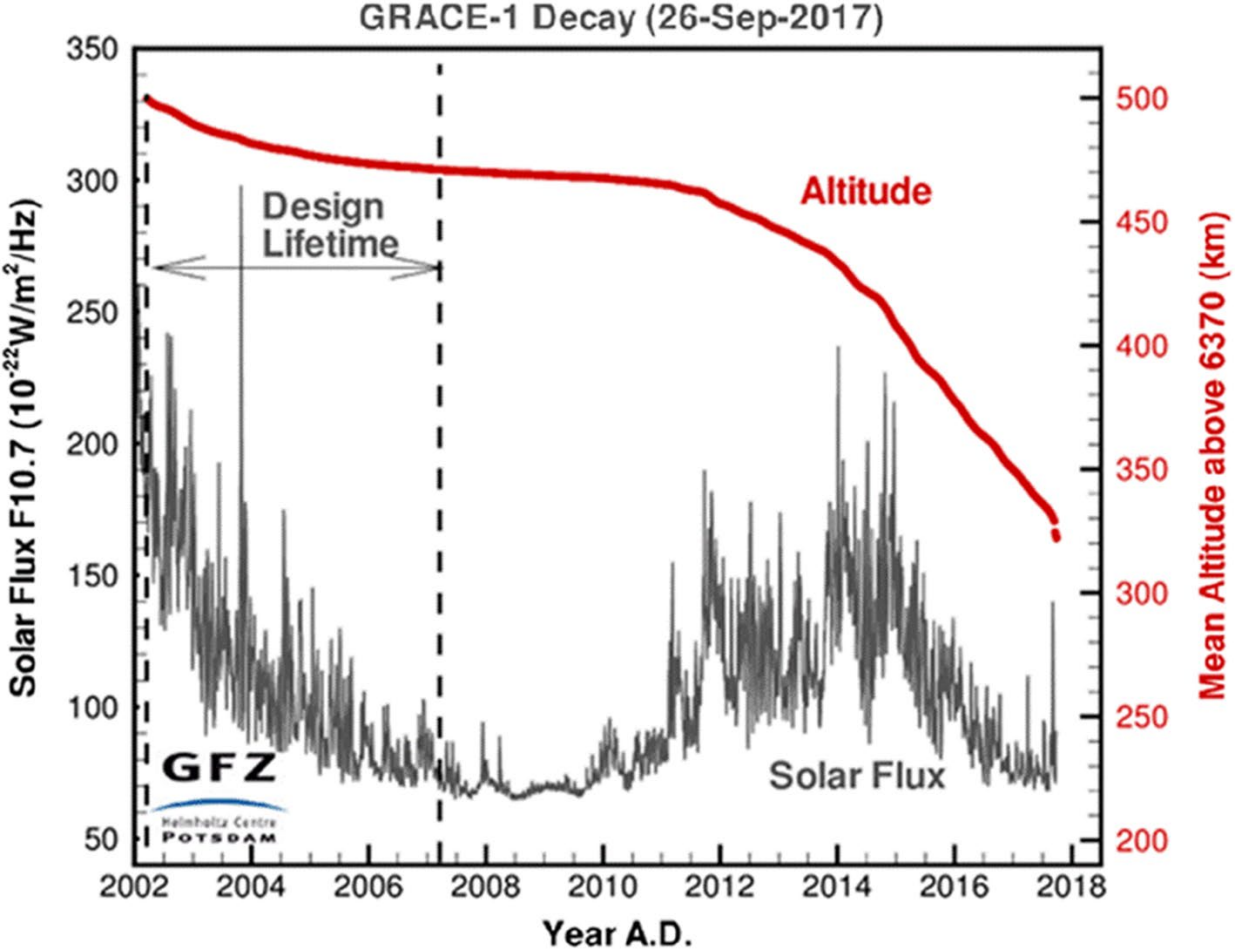
GRACE orbit altitude change between March 2002 and September 2017

During the 15-year mission period, which was three times longer than originally planned (Fig. 8), all instruments on board the GRACE satellites delivered almost continuously measurement and control data for satellite operation and monitoring of the instruments as well as for scientific evaluation. In September 2017, due to the age-related failure of a large number of battery cells on GRACE-2 and outgoing fuel, tandem operation had to be discontinued. This marked the end of the extremely successful active GRACE long-term mission. On 24 December 2017 GRACE-2 burnt up in the Earth's atmosphere. The twin GRACE-1 followed the same fate on 10 March 2018.

**Fig. 9:**
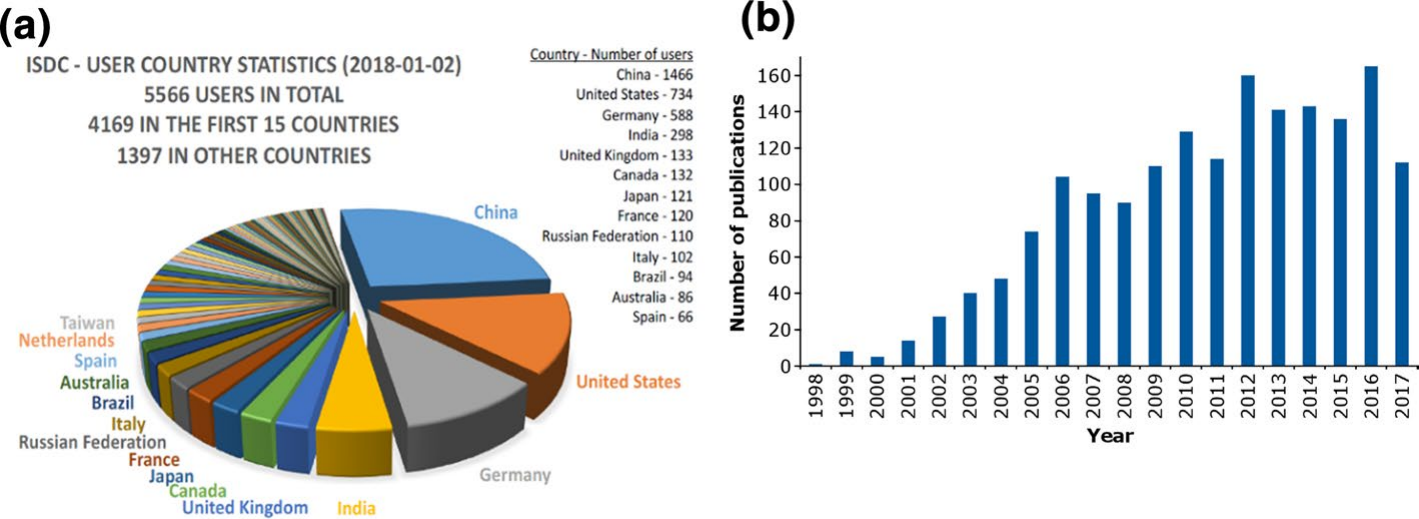
Statistics of worldwide registered users in the ISDC in early 2018 (left) and annual GRACE publications (right)

All measurement data obtained onboard GRACE were processed and archived in the GRACE Science Data System (SDS), jointly operated by JPL, CSR and GFZ, to form so-called level-0 (original raw observations) to level-2 (monthly gravity field models in terms of spherical harmonic coefficients) GRACE products. The archiving of the products and supporting documents was performed in the JPL Physical Oceanography Distributed Active Archive Center (PODAAC) and the Information System and Data Center (ISDC) operated at the GFZ. Both archives were continuously and automatically harmonized. Worldwide users can retrieve all GRACE data and gravity field products calculated by the SDS from there. Figure 9 shows the number of registered users at the ISDC as of 2 January 2018 and statistics of publications resulting from GRACE until the end of 2017. The most important characteristics of GRACE are summarized in Table 5.

**Table 5:** Characteristics of GRACE

GRACE	Gravity Recovery and Climate Experiment
Main instrument for gravity field recovery	Low Earth orbiting identical satellite pair with K-Band inter-satellite ranging system HAIRS, low–low SST
Other instruments	Spaceborne geodetic GPS receiver
Separation distance of satellite pair	220 ± 50 km
Orbit determination	Spaceborne geodetic GPS receiver
Orbit control	Laser retro reflector
Orientation in space	Two stellar sensor systems
Measurement of non-gravitational accelerations	Electrostatic Super-STAR accelerometer
Mission duration	17.3.2002–24.12.2017
Orbit height	Descending from an altitude of 500 km after launch to 345 km in September 2017
Orbit inclination	89° (quasi-polar)
Orbit eccentricity	quasi-circular

#### Time-Variable Gravity Field Models

The most important evaluation goal of the GRACE mission was the calculation and rapid provision of time-variable gravity field models. The SDS calculated monthly and weekly time-variable GRACE gravity field models almost without interruption from April 2002 to June 2017. These time series of the three evaluation teams, which are important for further interpretation and evaluation, were provided in the form of so-called GRACE Level 2 products, i.e. as a set of spherical harmonic coefficients describing the Earth's gravity potential for a certain time period and a spatial resolution. The time series has been reprocessed six times based on improved instrument data, background models or processing standards. These release versions are stored, together with helpful documentation, in the two GRACE archives ISDC and PODAAC.

Time series of monthly gravity fields are available in the versions RL01 to RL06. The time series GFZ RL06 (Dahle et al. 2019) covers, like all other SDS RL06 versions, the period April 2002 to June 2017 and consists of 163 monthly solutions. In some months, there were no gravity field solutions due to missing or non-nominal Level-1B instrument data, mainly caused by battery problems on GRACE-2 since early 2011. The RL06 models with a resolution of degree and order d/o 96 (about 208 km half-wavelength) represent gravity field variations caused by short-term, seasonal and long-term changes in continental and oceanic water masses, the mass of high mountain glaciers and ice sheets, crustal movements related to post-glacial land uplift and abrupt very strong Earthquakes, as well as errors or not modelled effects in the background models used.

**Fig. 10:**
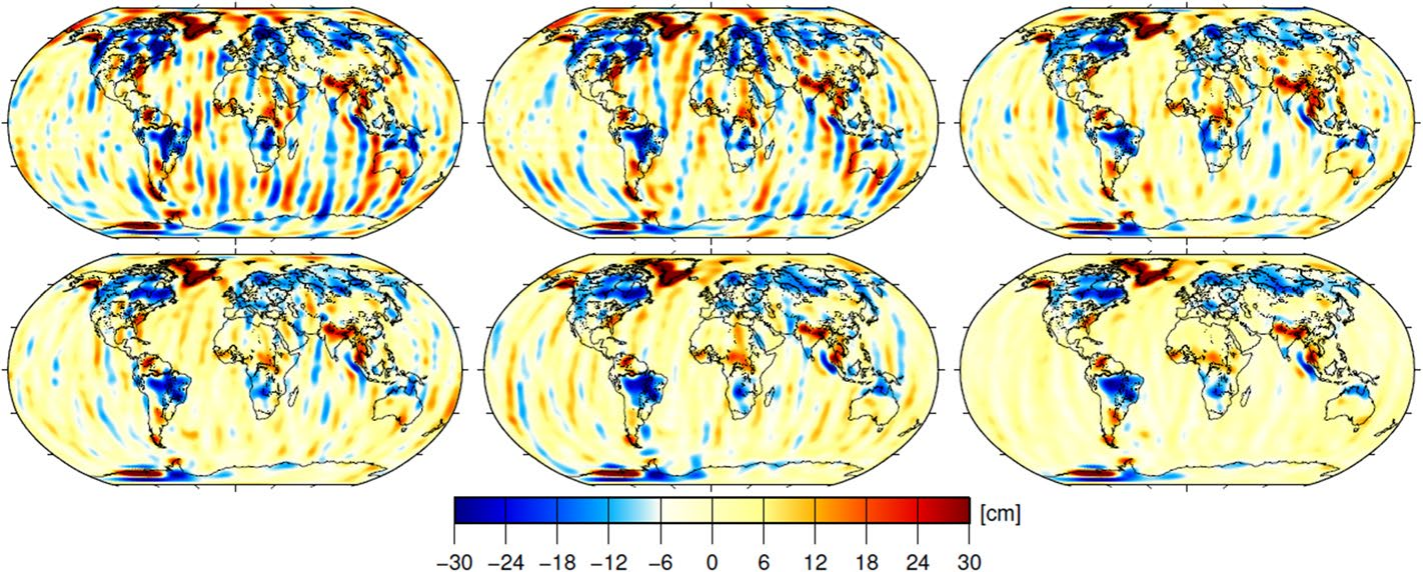
Gravity field anomalies expressed in terms of equivalent water height (EWH) with unit cm and DDK3 filtered for the month 2003/08 for all GFZ GRACE releases so far: RL01 (top left), RL02 (top middle), RL03 (top right), RL04 (bottom left), RL05 (bottom middle), and RL06 (bottom right). Figure is identical to Fig. 9 of Dahle et al. (2019)

Figure 10 shows mass anomalies in equivalent water heights for all GFZ GRACE releases exemplarily for the month August 2003 and without using the C_20_ coefficient, which cannot be precisely derived from GRACE measurements. All solutions in this figure show a more or less pronounced stripe pattern, a very characteristic phenomenon of the GRACE dual satellite configuration flying on a polar orbit with pure along-track sensitivity and undersampling of high-frequency signals in the monthly solutions. This phenomenon spurred intensive research into improvements of background modelling and spherical harmonic filter theory. To reduce the impact of this spatially correlated noise the solutions in Fig. 10 are de-correlated and smoothed by applying the non-isotropic DDK filter (Kusche 2009). The corresponding relative improvements in terms of wRMS over the ocean are as follows:

- RL01—> RL02: 14%
- RL02—> RL03: 24%
- RL03—> RL04: 4%
- RL04—> RL05a: 0%
- RL05a—> RL06: 41%.

This confirms remarkable improvements achieved with the GFZ RL06 reprocessing and also depicts that even after more than 15 years of the first instrument data release a substantial gain in the quality of monthly GRACE gravity field products is still possible thanks to reprocessing efforts of Level-1 instrument data, continuously improved background models, especially for tidal and non-tidal mass variations, and enhanced processing strategies.

In addition, time series of weekly gravity fields were formed by the GFZ for RL05 by solving subsets of the monthly normal equation systems corresponding to the division of the GPS weeks. They are characterized by a higher temporal resolution, but at the expense of a lower spatial resolution.

In addition to the SDS monthly up to weekly products mentioned above, time-variable GRACE gravity field solutions with different evaluation methods have been calculated and made available by e.g. the following processing centres in the last years:

- Mascon solutions from GSFC (Luthcke et al. 2013), JPL (Watkins et al. 2015) and CSR (Save et al. 2016)
- Models of mass transport from Delft Institute for Earth-Oriented Space Research (DEOS) (Liu et al. 2010)
- Monthly (Lemoine et al. 2007) and 10-day (Bruinsma et al. 2010) solutions combining GRACE and LAGEOS data from GRGS (Groupe de Recherches de Géodésie Spatiale) Toulouse
- Daily and monthly models from the Institute for Geodesy and Geoinformation (IGG) at the University of Bonn and the Technical University of Graz (Kvas et al. 2019)
- Monthly models from the Astronomical Institute (AIUB) of the University of Bern (Lasser et al. 2020)
- Monthly models by the Huazhong University of Science and Technology, Wuhan, PR China (Zhou et al. 2016) or the Wuhan University, PR China (Guo et al. 2017)
- Monthly models by the Leibniz University Hannover (Koch et al. 2020)

#### Static Gravity Field Models

In addition to the time-variable gravity field models, the SDS team and various groups in Europe, the USA and China computed static gravity field models. For these static gravity models, i.e. models averaged over longer periods of time, a distinction was made between *GRACE-only*, *satellite-only* (from a combination of GRACE, GOCE, CHAMP and/or LAGEOS data) and *combined* models, in which additional terrestrial gravity data were included. From the great number of calculated models only some typical examples from the entire analysis period are listed:

- First and more recent "GRACE-only" gravitational field models (Reigber et al. 2005; Tapley et al. 2005; Jäggi et al. 2010; Chen et al. 2018; Mayer-Gürr et al. 2018).
- "Satellite-only" gravity field models complete to high degree and orders and computed from GOCE, GRACE and LAGEOS data (Pail et al. 2010; Bruinsma et al. 2010; Farahani et al. 2013; Zhou et al. 2019), and
- “High-resolution combination” gravity models, complete to very high degree and orders and computed from GOCE, GRACE, LAGEOS and surface gravity data (Förste et al. 2014; Zingerle et al. 2020).

All gravity field models are available for download in the ICGEM (International Center for Global Earth Models, http://icgem.gfz-potsdam.de) database of the GFZ. The ICGEM offers, besides further static and time-variable models, also web-based visualizations of the models, as well as interactive service offers for the computation of various gravity field quantities for further use (Ince et al. 2019).

#### Climate-Relevant Applications

With the systematic generation of accurate, monthly gravity field models from the globally acquired GRACE measurement data over a 15+ years mission period, a completely new remote sensing method has been established to detect mass changes in the geosphere from space. In an impressive wealth of scientific publications geodesists, geophysicists, glaciologists, hydrologists and oceanographers worldwide have carried out a wide range of investigations and analyses, with recourse to GRACE data, GRACE-SDS products or own GRACE evaluations. They deal with the variation of continental water storage, heat and mass exchange between ocean and atmosphere, general ocean circulation and changes in the mass budget of the Greenland and Antarctic ice, but also seafloor currents and mass distribution in the Earth's interior. With an online search for “GRACE mission” using a standard search engine such as http://scholar.google.com over 1.77 million relevant results can easily be found.

A significant part of these publications deals with GRACE results concerned with climate-relevant phenomena and human overuse of natural resources (Rodell et al. 2018). Of the whole wealth of wonderful results, only a few examples are given here as examples, some of which have also found their way into the analyses of the Intergovernmental Panel on Climate Change (IPCC) in its fifth Assessment Report AR5 of 2014 (Climate Change 2013, 2014).

*Groundwater Monitoring* Groundwater stored in soils and water-bearing rock strata (aquifers) could hardly be measured on a global scale so far. It has been shown in an impressive way that smallest mass changes observed with GRACE can now help to document the overexploitation of groundwater resources. Thanks to GRACE data, more and more aquifers have been identified over the last ten years that are being emptied by humans faster than they can replenish themselves. In 2015, a survey was published that showed that one third of the world's largest groundwater basins are dramatically overused (Richey et al. 2015).

*Flood Events and Crisis Management* In the framework of the EGSIEM (European Gravity Service for Improved Emergency Management) project, which was funded by the EU Horizon2020 program for the period 2015–2017, the GFZ and Graz University of Technology calculated daily solutions in near-real time (< 2 days) and derived moisture indicators from them. These are needed to predict the origin and development of flood events in large river systems. The derived moisture indicators were successfully used operationally in a three-month test phase (April–June 2017) at the satellite-based Crisis Information System (ZKI) of DLR. Based on historical flood events, it could also be shown that in some cases the advance warning times could be reduced to 6 weeks, e.g. in the case of the Danube floods of 2006 and 2010 (Gouweleeuw et al. 2018; Jäggi et al. 2018). In addition, the daily moisture indicators derived from GRACE data were implemented in a pre-operational manner in the forecasting system of the Global Flood Awareness System (GloFAS), which was jointly developed by the European Commission and the European Centre for Medium-Range Weather Forecasting (ECMWF).

*Polar Ice Sheets* Antarctica is an extremely inhospitable place to collect in-situ data, and Greenland is comparably problematic. Nevertheless, it is very important to know how fast the total mass of ice sheets changes in these areas in order to better understand the fluctuations in sea level worldwide. Researchers working on the cryosphere were among the first pioneers in the use of GRACE data. It quickly became clear that the mass loss of both Greenlandic and Antarctic ice is far more dramatic than previously thought. Expressed in figures, Greenland has lost 280 billion tonnes of ice per year since the start of GRACE, and Antarctica around 120 billion tonnes (Velicogna et al. 2014). Sasgen et al. (2010) have shown how the seasonal fluctuations in snowfall and the resulting increase in mass on the Antarctic Peninsula are related to the strength of a low-pressure system over the Amundsen Sea. This low- pressure system in turn is linked to the tropical La Niña phenomenon (the counterpart of El Niño). GRACE data have thus made it possible for the first time to quantify the effect of atmospheric "teleconnections" that link the climate of the tropics even with remote regions such as Antarctica.

*High-Mountain Glaciers* GRACE data also indicate the mass loss of glaciers in many high mountain regions of the world. This loss of water is accompanied by a threat to the water supply of the areas downstream of the mountains and the danger of glacial lake outburst floods (GLOFs). An international team of researchers (Farinotti et al. 2015) for example estimated on the basis of GRACE data that the Tian Shan high mountains in Central Asia are currently losing twice as much ice annually as the whole of Germany consumes in terms of water per year. Coupled with a glaciological model, the data showed that half of all glacial ice in the Tian Shan could have disappeared by 2050.

*Sea Level and Ocean Dynamics* The seawater warms up and therefore expands. In addition, there are increased inflows from the glacier regions and ice sheets of the Earth. Both contribute to the rise in sea levels worldwide. Although high-precision sea-level measurements have been available since 1992 via the US-French Topex-Poseidon and the subsequent Jason satellite altimetry missions, they only show the total height changes of the sea surface. To find out whether the (temperature-related) expansion of water, or melting ice, or the influx of water from land has a greater effect on these changes, one has to study the mass distribution of water, as shown for example for the Antarctic Circumpolar Current with GRACE data by Bergmann and Dobslaw (2012).

A comprehensive overview of contributions of GRACE data for a better understanding of climate change is provided in Tapley et al. (2019).

### GOCE

#### The Prehistory

Gravitational gradiometry is the measurement of gradients of the three components of the gravitational vector, or in other words, of second derivatives of the gravitational potential. One could call the geodetic torsion balance developed by the Hungarian physicist and geodesist Lorand Eötvös the first gradiometer (Selényi 1953). The Eötvös torsion balance was used in geodesy and exploration geophysics for many years.

Already in the sixties and seventies of the last century there were proposals for the development of a satellite gravity gradiometer, it is referred to for example (Carroll and Savet 1959; Diesel 1964; Forward and Miller 1967; Forward 1972; Savet 1969). This resulted in several alternative lines of development (Wells 1984), some of which also found their way into the plans of NASA (1972) and NRC (1979). Fundamental work on the theory of geodetic satellite gradiometry goes back to (Moritz 1968, 1986; Meissl 1971, Marussi 1979, 1984; Marussi and Chiaruttini 1985; cf. Rummel 1979, 1986). While NASA, GFZ and some other German institutions finally concentrated on the realization of a satellite-to-satellite tracking mission in low-low mode, the gradiometry concept was pursued on the European side within the framework of ESA. Based on the positive experiences with the accelerometer CACTUS on the French satellite mission CASTOR (D5B) (Bernard et al. 1985), a gradiometer mission called GRADIO and studied at CNES (Balmino et al. 1984, 1985) was proposed for the ESA science program Horizon 2000. The mission concept for GRADIO was also the basis for the mission proposal ARISTOTELES, a combined measurement of the Earth's magnetic field and gravity field (ARISTOTELES 1989, ESA 1991a, b). Neither GRADIO nor ARISTOTELES made it; the required technology was very challenging and ESA’s program was dominated by remote sensing. After the definition of ESA's own Earth science satellite program, the "Living Planet Program" in the 1990s, it was possible to push forward a pure gravity gradiometry mission, building on the experience with GRADIO and ARISTOTELES. The mission proposal GOCE (ESA 1999; Rummel et al. 2002; Johannessen et al. 2003) was chosen by scientists and ESA delegates in a multi-stage selection process between 1996 and 1999 as the first Explorer mission of this program.

#### GOCE: Mission Objectives and Principle

GOCE was the first satellite of the European Space Agency ESA's "Living Planet" Earth science program. It was launched on 17 March 2009 by a Russian launch vehicle from Plesetsk. The aim of this mission was to measure the gravitational field of the Earth as detailed and accurate as possible.

**Fig. 11:**
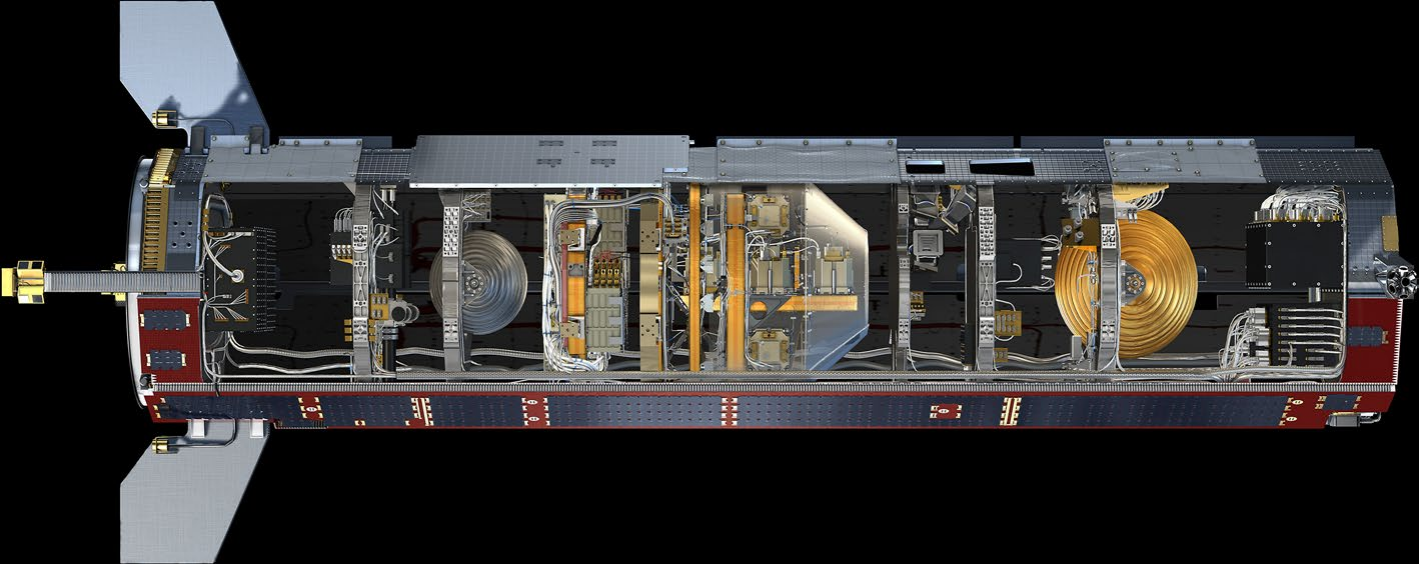
The inner workings of the satellite GOCE—in the centre the gravitational gradiometer, immediately to the right the star sensors and in the next segment to the right the GPS receiver of European design (*Source*: ESA)

The abbreviation GOCE stands for "Gravity and steady-state Ocean Circulation Explorer". Figure 11 shows the interior of the satellite together with its sensors. The mission was mainly driven by geodesists, and even today the evaluation of the measured data is mainly in the hands of geodesists. The project also provided important data for geodesy. However, the scientific objectives are primarily in the fields of oceanography and geophysics, see Sect. 3.3.4. This underlines the fact that geodesy provides very relevant contributions to the Earth sciences in general and to climate research in particular.

GOCE's mission objective was to measure the global Earth's gravitational field in unprecedented detail. The measurement setup was designed to resolve spatial structures of the Earth's gravitational field up to a spatial extent of about 100 km half wavelength. This may seem rather modest compared to the achievable pixel size of modern imaging techniques. However, for a gravity field measurement system in a satellite this is almost the limit of what is possible. The desired accuracy was 1 millionth of the Earth's gravity (= 1 milliGal) or a geoid height accuracy of 1 to 2 cm. In order to achieve the desired accuracy and resolution an extremely low, i.e. near-Earth orbit, of only 255 km above the Earth's surface was chosen. GOCE used also an air drag compensation system and was the first test of the principle of gravitational gradiometry in a satellite. The complete mission design is described in Drinkwater et al. (2007).

#### Measuring Principle

GOCE used two complementary measuring systems to determine the gravitational field of the Earth. With a geodetic GPS receiver of European design, the orbital trajectory was measured with centimetre accuracy. The purely geometric approach used here—known as kinematic orbits—is based on measurement series of both the GPS code and the phase of the GPS carrier waves. From the calculated trajectory the large-scale part of the Earth's gravity field was derived (Bock et al. 2011).

**Fig. 12:**
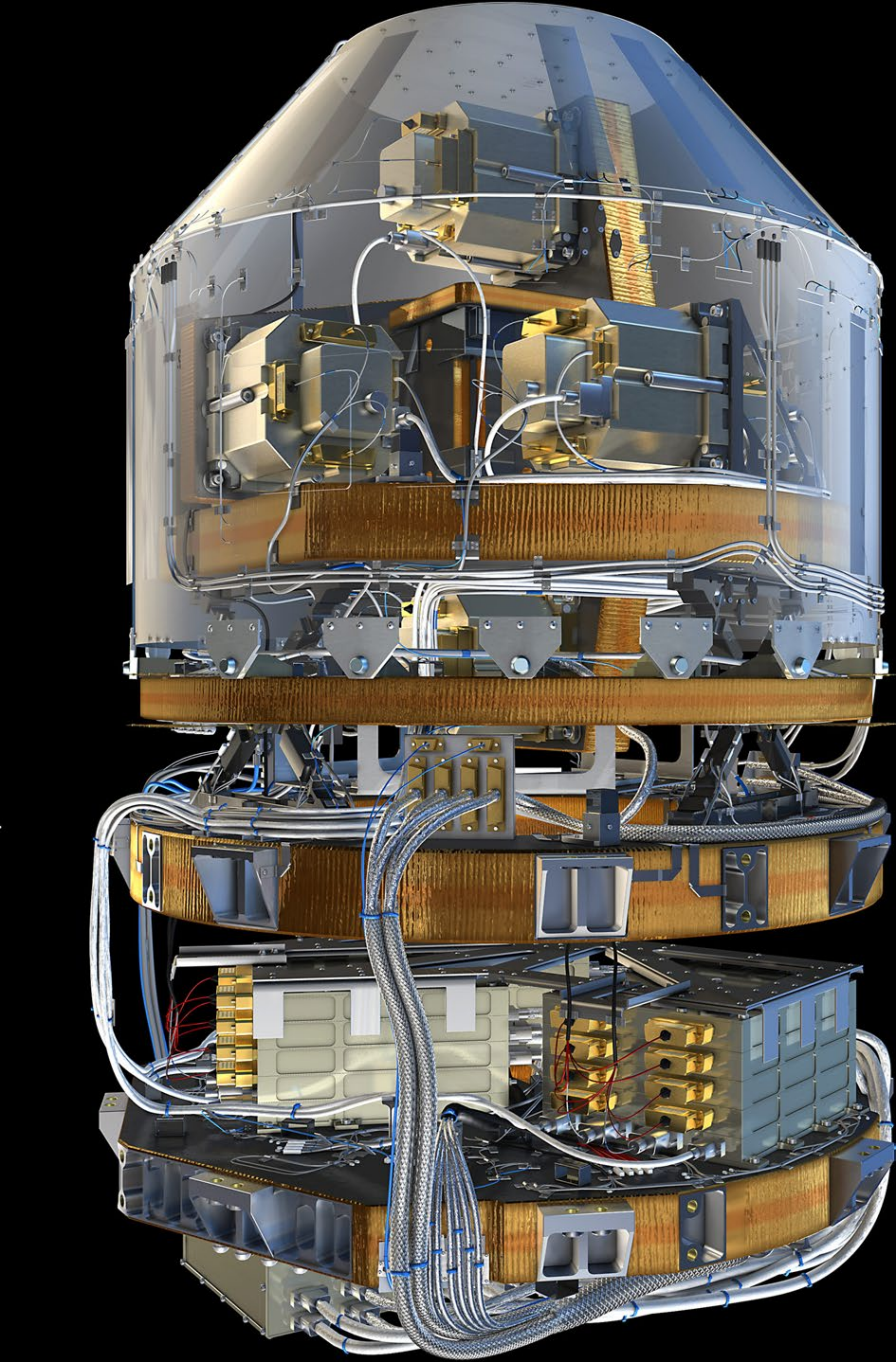
GOCE gravity gradiometer—in the upper part, three of the six accelerometers can be seen, each of them is mounted at the end of three orthogonally arranged axes. The lower part shows the electronic read-out system ( *source*: ESA)

The details of the gravitational field were determined from the measurements of the gravitational gradiometer, see Fig. 12. The GOCE gradiometer consists of three arms arranged perpendicular to each other, each 50 cm long, with a triaxial accelerometer attached to each end. Each of the three arms with two of these accelerometers forms a uniaxial gradiometer. The x-axis of the gradiometer pointed in the direction of flight, the y-axis orthogonal to the satellite orbit plane and the z-axis approximately radially outwards from the centre of the Earth. The centre of the instrument was located in the centre of mass of the satellite. Thus, the accelerometers measured accelerations in *x*-, *y*- and *z*-direction at six points in the satellite, arranged symmetrically to the satellite centre, i.e. at the ends of the three axes. The signal is composed of a gravitational component and a rotational component. The rotational component is a consequence of the angular movement of the satellite (and its instruments) along its orbit relative to the inertial space. The gravitational component corresponds to the gravitational acceleration at the location of the accelerometer relative to that at the mass centre of the satellite. Both components are very small, typically not greater than one millionth of the Earth's gravitational pull, i.e. of "g" on the Earth. This is why we also speak of "microgravity". For this approach, extremely sensitive accelerometers had to be developed (Touboul 2003). This was the contribution of ONERA, a French laboratory developing space instrumentation in Paris. As written previously the accelerometers used for CASTOR, CHAMP and GRACE also originate from ONERA (Touboul et al. 1999a, 1999b). Each of the six GOCE accelerometers consists of a measuring chamber in which a 320 g rhodium-platinum plate (4 cm × 4 cm × 1 cm) is kept levitated by an electrostatic capacitive feedback system; the feedback signal is proportional to the rotation/gravity signal. Since these sensors are built in the laboratory on Earth under the influence of the Earth's gravity, one axis of each accelerometer had to be designed much more robustly and therefore less accurately in order to electrostatically compensate for the force of the Earth's gravity on the sample mass, see Marque et al. (2010).

The sum or difference is taken from the measured rotation/gravity signal of the two accelerometers at the ends of each axis in *x*-, *y*- and *z*-direction, respectively. Thus, for example, along the *x*-axis, i.e. in the direction of flight, the components $\{xx\}$, $\{xy\}$ and $\{xz\}$ are measured as differences and thus from all three axes a total of nine components is measured. In principle, one would like to have the six gravity gradients ${V}_{xx}$, ${V}_{xy}, {V}_{xz}$, ${V}_{yy}$, ${V}_{yz}$ and ${V}_{zz}$ as well as the three angular rates ${\omega }_{x}{, \omega }_{y} and {\omega }_{z}$. However, since one axis of each accelerometer is less accurate, only the gradients ${V}_{xx}$, ${V}_{xz}$, ${V}_{yy}$ and ${V}_{zz}$ as well as the—$\mathrm{angular rate }{\omega }_{y}$ the rotation rate of the satellite on its orbit around the Earth—can be derived with the highest accuracy. But this is sufficient: from each of the four gravitational gradients, the global gravitational field of the Earth can already be calculated. In addition, the so-called LAPLACE equation gives the condition for three of the gradients: ${V}_{xx}+{V}_{yy}+{V}_{zz}=0$, which must be valid at every point along the satellite orbit, a very important quality control from which an accurate picture of the level of the measurement noise could be derived. The high gradiometric accuracies were achieved in a measuring band between 0.005 and 0.1 Hz. The rotation rates are determined from the gradiometer measurements in combination with the star sensor measurements. The sum signals of the measured accelerations correspond to the non-gravitational forces acting on the satellite body, especially the frictional influence of the residual atmosphere still present at this orbital altitude. The sum signal in flight direction of GOCE was used in a feedback loop to compensate for the "air resistance" acting on the satellite body with very sensitive ion thrusters. The orbital motion in flight direction was therefore purely gravitational. Only in this way was it possible to orbit GOCE around the Earth at an altitude of only 255 km for the entire mission period and, in the final phase, even to lower it in several stages to an altitude of only 224 km, see Table 6**.** Like the gradiometer itself, this "drag compensation system" was also a novelty and had never been tested on any geoscientific satellite before. The inclination angle of the orbital plane with respect to the equatorial plane was 96.7°. This setting is called sun-synchronous, because for this inclination the orbital plane—and thus the satellite—remains facing the sun during the whole mission by a slow gyroscopic movement in space. The advantage is an optimal supply of solar energy, the disadvantage is that an area with an aperture angle of 6.7° at the north and south pole of the Earth remains without data coverage, see also (Floberghagen et al. 2011; Frommknecht et al. 2011). The most important characteristics of GOCE are summarized in Table 6.

**Table 6:** Characteristics of GOCE

GOCE	Gravity and steady-state Ocean Circulation Explorer
Main instrument	Triaxial gravitational gradiometer
Other instruments:	
Orbit determination	Spaceborne geodetic GPS receiver
Orbit control	Laser reflector
Orientation in space	Three star-sensors
Compensation for air resistance	Ion engines
Mission duration	17.3.2009–11.11.2013
Orbit height	255 km Starting on 1.8.2012 lowering of the orbit -height in 4 steps by 9 km, 6 km, 5 km and 11 km to a height of 224 km
Orbit inclination	96.7° (sun-synchronized)
Orbit eccentricity	Quasi-circular

Only the perfect interaction of all the measuring systems on board GOCE did allow the extreme accuracy of the gravity gradiometer to be used to the fullest. More than 40 European companies were involved in the construction of the complete satellite system. All sensors had survived the satellite launch unharmed and were working flawlessly; a truly outstanding engineering achievement.

#### Gravity Field Models

After each time segment of 61 days, GOCE had achieved a uniform, global data coverage that was sufficient for the calculation of a gravity field model. Between a few of these repeat cycles the gradiometer was calibrated. ESA published five editions of the gravity field models during the mission, each with an increasing data volume (Brockmann et al. 2014; Bruinsma et al. 2014). The models are presented as sets of dimensionless spherical harmonic coefficients up to a maximum degree of development. There is a model series TIM (for "timewise"), which is based exclusively on GOCE mission data, and a model series DIR (for "direct"), which also includes GRACE data and SLR data. The methods are described in Pail et al. (2011), their characteristics are summarized in Table 7. Finally, so-called SPW models (for "spacewise") were published, which are offered as global geoid or gravity anomaly grids. An overview is given in Table 8. It is also referred to the special issue of the J. Geodesy, volume 85, number 11 November 2011.

**Table 7:** Characteristics of gravity field models DIR5 and TIM5

	DIR5	TIM5
Max. degree of spherical harmonic series development Degree/order (d/o)	300	280
Data volume	01.11.09–20.10.13	01.11.09–20.10.13
Gravity Gradients	${V}_{xx},{V}_{yy},{V}_{zz},{V}_{xz}$	${V}_{xx},{V}_{yy},{V}_{zz},{V}_{xz}$
Filter method	Bandpass	ARMA per data segment
GOCE SST (GPS)		Short Arc Method (d/o 150)
GRACE SST (K-Band)	Years 2003–2012 GRGS RL03 (d/o) 130)	
LAGEOS 1 & 2 (SLR)	1985 – 2010	
Regularization	Spherical cap related to GRACE & LAGEOS Kaula for (d/o > 180)	Kaula (for a segment near the zonal coefficients and for d/o > 200)

**Table 8:**
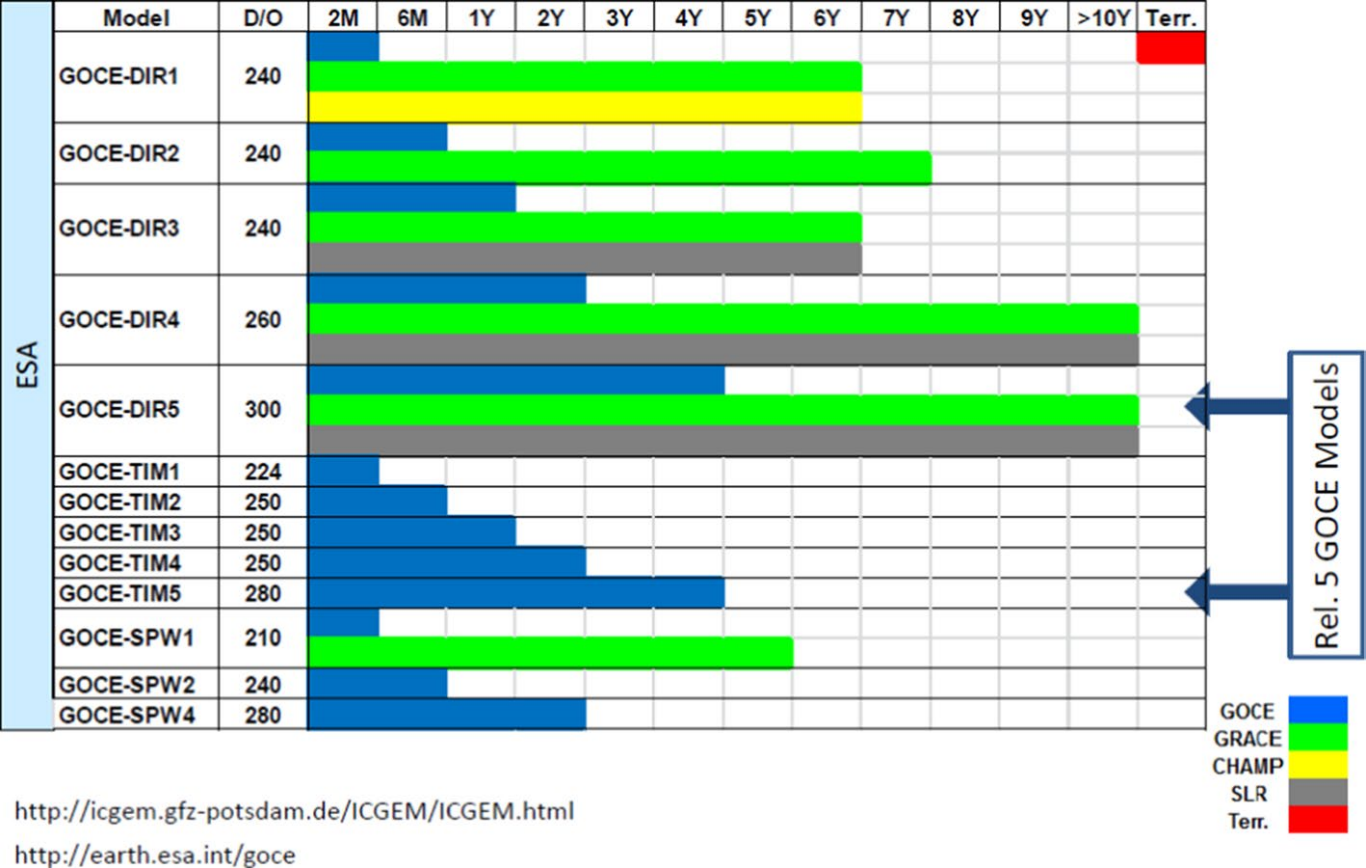
Overview of the ESA GOCE gravity field models of the DIR, TIM and SPW series

#### Scientific Results

The following is a very brief summary of some results of the geophysical, oceanographic and geodetic use of GOCE. Reference is made to Rummel (2020).

Gravity or geoid anomalies are a measure for the imbalance of the Earth masses in the crust, lithosphere and upper mantle (Alvarez et al. 2014; Bouman et al. 2016; Braitenberg 2015; Ebbing et al. 2014; v/d Meijden et al. 2015; Panet et al. 2014). New models of the Mohorovicic (Moho) discontinuity were derived from the gravity field models of GOCE. The Moho discontinuity is the boundary surface between the Earth's crust and the mantle and corresponds to the depth of the isostatic mass balance according to the Airy model. A comparison with the seismically derived depth of this interface allows conclusions about the actual compensation mechanism. Detailed investigations were carried out for South America, parts of Africa and Asia, and Antarctica (Reguzzoni and Sampietro 2015; Shin et al. 2015; Li et al. 2017). For these regions, GOCE demonstrated that the gravity data available before GOCE is faulty and incomplete (Hirt et al. 2011; Yi and Rummel 2014). In the central part of the Himalayas and in the northern Indian border zone, GOCE gradient data were used for the first time to determine plausible values for the elastic thickness of the lithosphere (McKenzie et al. 2014). Also new is the now available gravity and geoid model of the Antarctic. It provides important information about the geological development, which was previously hidden under an ice sheet several kilometres thick (Ferraccioli et al. 2011; Fretwell et al. 2013; Hirt 2014; McKenzie et al. 2015). The specific value of gravity gradients is shown in Ebbing et al. (2018), Sebera et al. (2018), Plasman et al. (2020).

Contrary to the original expectations, the combination of GOCE models with GRACE gravity time series succeeded in increasing their spatial resolution of the monthly solutions (Fuchs et al. 2013; Garcia et al. 2013). The gradiometer sum signal and the feedback signal for the compensation of the atmospheric drag also offered insights into atmospheric density and atmospheric winds (Doornbos et al. 2013; Gasperini et al. 2017; Liu et al. 2016; March et al. 2017).

For oceanography, GOCE provided for the first time a detailed global geoid model. Satellite altimetry, another highly successful geodetic satellite technique, has been providing series of measurements of the actual and mean sea level without interruption for more than 20 years. The difference between altimetric sea level and geoid height is the dynamic sea surface topography, i.e. the actual or mean elevation of the sea surface above or below the geoid. The topography values are a few decimetres only, maximum values of 1–2 m are only reached in the centres of large circulation systems, for example in the Gulf Stream, Agulhas Current, Kuroshia Current or Circumpolar Current. Only due to the exceptionally high quality of altimetry and GOCE geoid model it became possible to reconstruct an accurate global image of the ocean topography with this purely geodetic approach, independent of ocean models. Via a simple mathematical operation, the global vector field of the geostrophic ocean currents follows from the ocean topography. Ocean topography and current velocities are new input variables in numerical ocean circulation models. See for example Albertella et al. (2012), Bingham et al. (2011), Haines et al. (2011), Jancjic et al. (2012), Knudsen et al. (2011), Rio et al. (2013), Rio et al. (2014), Woodworth et al. (2015). Both the calculations of mass transport and heat transport in the oceans are thus improved. They also represent an important geodetic contribution to the ongoing climate debate.

Analogous to the approach used in the ocean areas to calculate marine topography from the difference between sea level and geoid height, topographic heights on land are derived from "GPS levelling". GPS levelling, or (in view of several operational satellite navigation systems) more correct GNSS levelling, is the calculation of physically relevant working heights from the difference of ellipsoidal height from GNSS and geoid height. Operational heights can be normal heights, as in the Federal Republic of Germany, orthometric heights, as in Switzerland, or geopotential numbers, as in the adjustment of the European height network. In the medium term, the GNSS levelling method will replace classical levelling as the primary method for determining height. In some countries corresponding decisions have already been made. Despite the very high accuracy of classical levelling over short distances, the advantages of GNSS levelling outweigh the disadvantages: In countries with a well-developed geodetic infrastructure, GNSS levelling is already sufficiently accurate for all applications, free of systematic deformation over large areas, cost-effective and very efficient. In addition, time series are generated at GNSS permanent points, with which possible changes in height over time can be detected. Until today, national and regional height systems refer to the mean sea level of the respective reference point; the official heights of the Federal Republic of Germany (heights above sea level), for example, refer to the reference level of Amsterdam (= Normaal Amsterdams Peil (NAP)). The reference level of the different height systems varies from country to country. This results in off-sets between the height systems of the individual countries in the centimetre to decimetre range (Gruber et al. 2013; Woodworth et al. 2012). Within Europe, a standardization with conventional levelling would be feasible at any time; however, height systems separated by sea areas cannot be compared by classical levelling. The heights calculated from GNSS levelling in combination with the geoid model from GOCE refer to a globally uniform geoid surface. Corresponding steps to recalculate the height networks are already being taken in the USA and Canada. This will result in three things: any differences in level between the various national height systems will be detectable, all operational heights can be standardized, and existing systematic deformations of the height networks will be uncovered and can be eliminated. As shown in Gruber et al. (2013), this approach allows a global comparison of mean sea levels of the oceans free of systematic deformations. A comparison of mean sea levels at tide gauges of the seas bordering on Central Europe with large-scale classical precision levelling was already pursued by Helmert (Börsch et al. 1891; Seibt 1883). The geoid model used for GNSS levelling should be based on the final results of the GOCE mission. At the same time, it should be the best possible combination, on the one hand with the other satellite gravity field information (GRACE, LAGEOS 1&2) and on the other hand with all terrestrial and altimetric gravity anomalies available worldwide. Existing data sets from classical levelling could still be used to improve the accuracy over short distances (Gerlach et al. 2016).

### GRACE Follow-on Mission

With a mission duration of more than 15 years GRACE has delivered data three times longer than originally planned. This underlines the excellent work of Airbus Defense & Space (D&S) GmbH, which built the two satellites in Germany on behalf of NASA/JPL. In addition, the operations team, consisting of DLR/GSOC, JPL, CSR, Airbus D&S and GFZ, which together monitored the daily performance of the satellites and their instruments, did a great deal to extend the mission duration as long as possible. Already since 2011, the batteries on both satellites showed clear signs of ageing, and the core instruments had to be switched off regularly for limited periods of time. By October 2017, the mission lifetime was finally over, as the satellite tandem had used up all its fuel supplies. The last monthly gravity field could be calculated for June 2017.

As described in Sect. 2.3.5, the mass transports derived from GRACE data are of enormous benefit for the investigation of many climate-relevant phenomena (Tapley et al. 2019). But because effects of climate change, natural variability, and anthropogenic influences in Earth’s water systems can only be distinguished with necessary reliability by means of long time series (if possible over several decades), NASA and GFZ had already decided in 2011 to realize a follow-up mission, named GRACE Follow-On (GRACE-FO), again in an American-German partnership (Flechtner et al. 2016). As with the GRACE mission, the Jet Propulsion Laboratory (JPL) commissioned the construction of the satellites from Airbus D&S GmbH, ordered the accelerometers from ONERA and the star sensors from DTU, and contributed the HAIR SST system and the SGPS receivers. Germany, under the responsibility of the German Research Center for Geosciences GFZ, has provided the launch vehicle, the mission operations for the nominal mission lifetime of 5 years, and participates in the joint SDS. Additionally, GFZ has provided Laser Retroreflectors for both satellites. The NASA/GFZ partnership is laid down in a Memorandum of Understanding, the roles and responsibilities in the joint project in a Cooperative Project Plan.

**Fig. 13:**
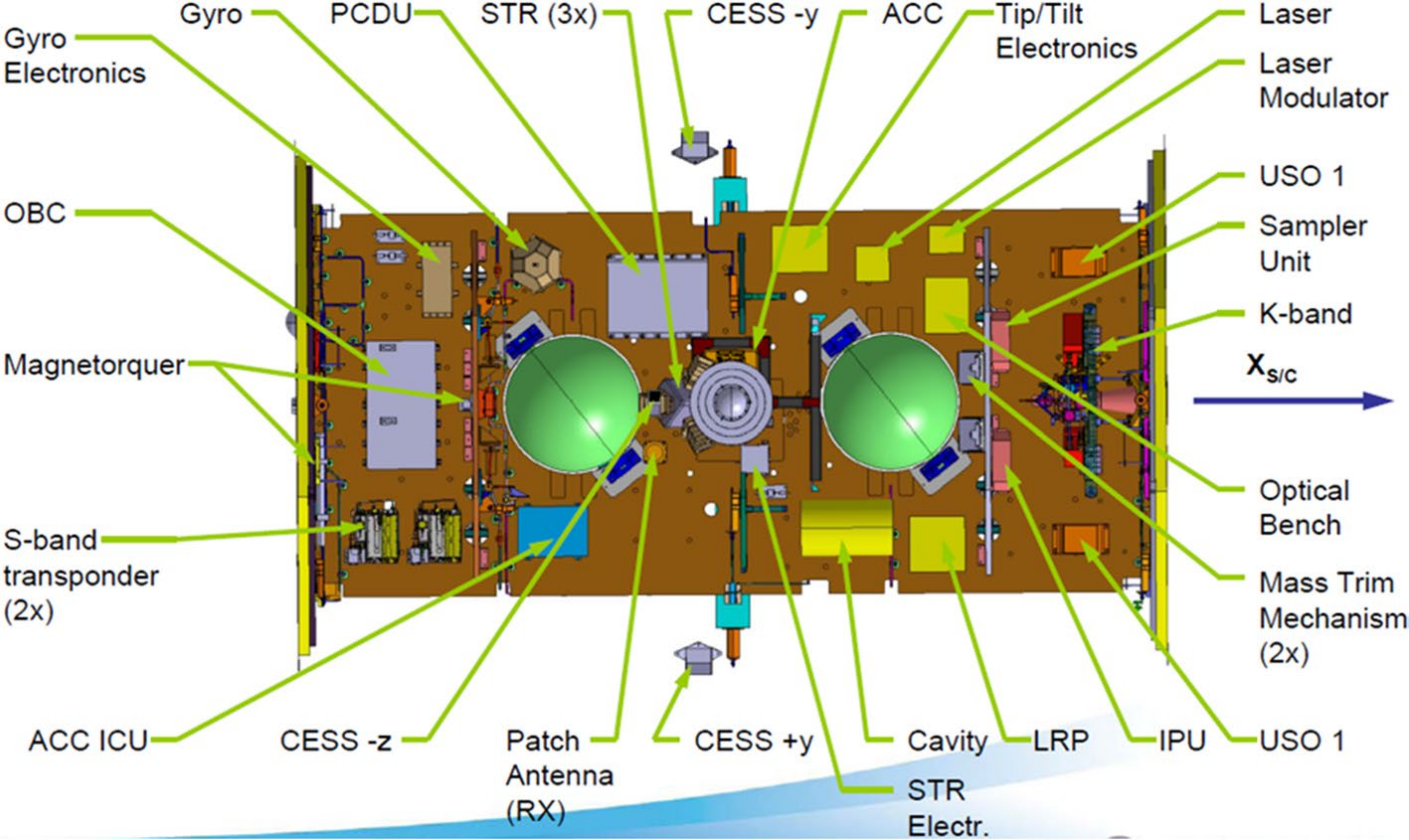
GRACE-FO interior

The GRACE-FO satellites (Fig. 13) had successfully completed all construction phases and environmental tests by November 2017. They were then transferred to the launch site at Vandenberg Air Force Base (VAFB) in California in December 2017, where they were prepared together with five satellites of the Iridium-Next series for a joint launch with a Falcon-9 rocket of the US company SpaceX.

**Fig. 14:**
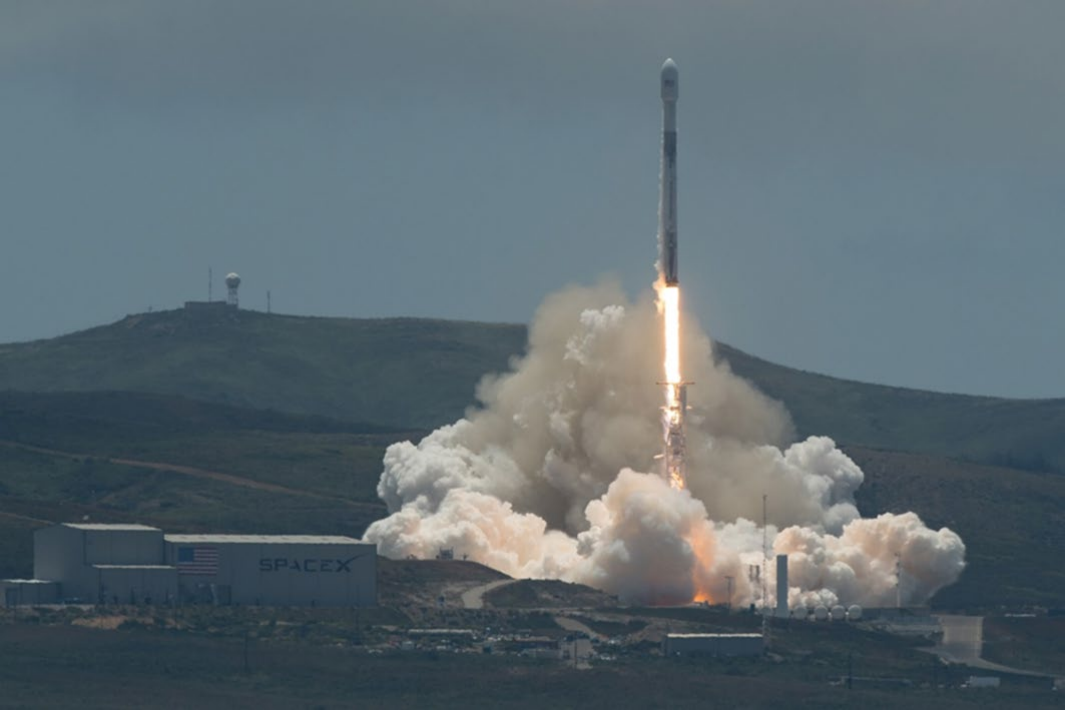
Successful launch of the GRACE-FO mission on 22 May 2018 (Photo SpaceX)

The launch took place successfully on 22 May 2018 (Fig. 14). The subsequent Launch and Early Operations phase was completed in less than four days and after about two weeks all instruments were already in operation. During the In-Orbit Check Out (IOC) phase, the quality of the observations of the different instruments was characterized in detail, all necessary calibration-manoeuvres were performed and the first gravity fields were generated. The IOC phase lasted until 28 January 2019 due to the necessity of changing the instrument processing unit (IPU) of the microwave instrument on the second satellite to its replacement unit. The mission is currently in its operational science phase. A total of 24 monthly gravity field models have already been generated in the SDS up to and including September 2020, all of which meet the quality requirements for these products defined prior to the mission (Landerer et al. 2020).

**Fig. 15:**
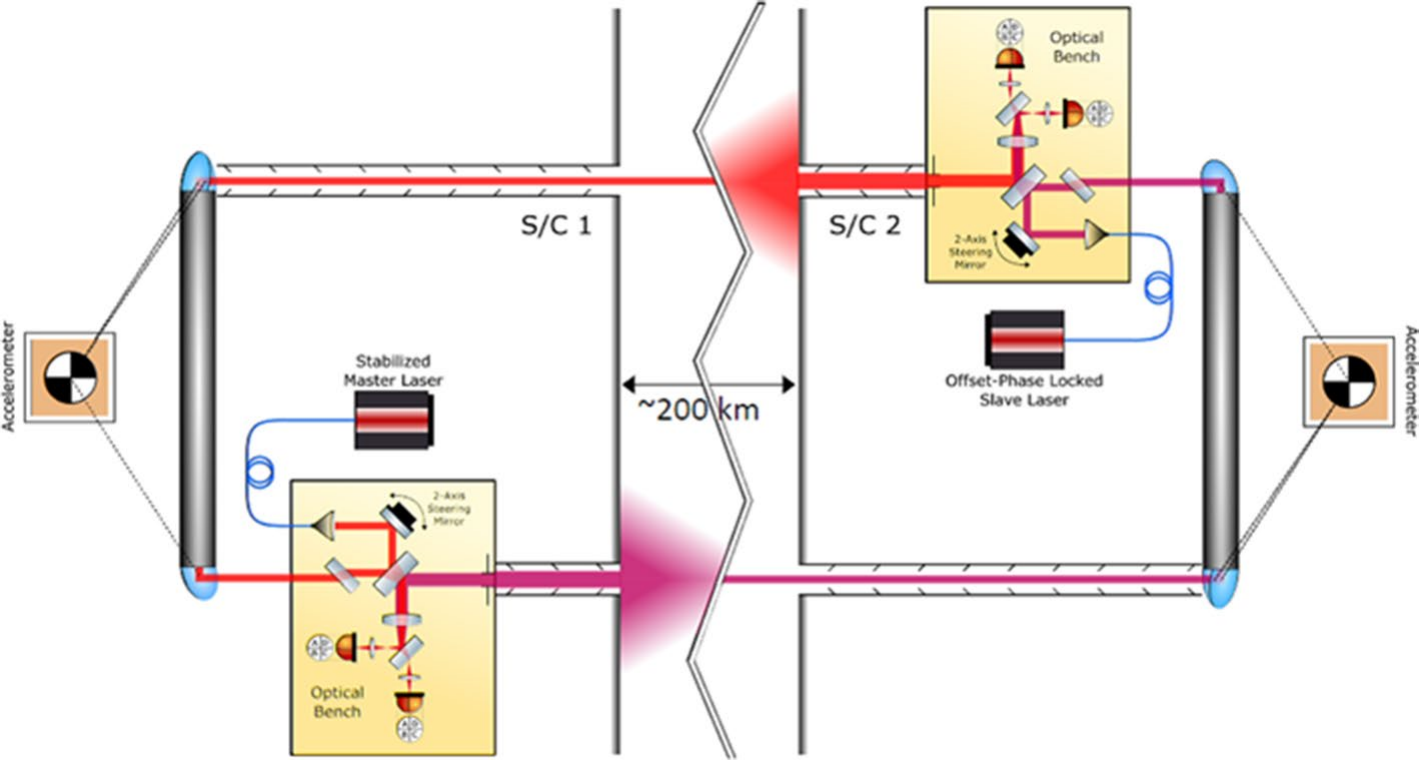
Schematic structure of the GRACE-FO laser-ranging interferometer (from Sheard et al. 2012)

As an important extension of the instrumentation very similar to that of GRACE, GRACE-FO flies a novel Laser Ranging Interferometer (LRI) as a demonstration payload for future gravity field missions. The LRI (Fig. 15) is a joint development where the electronics and the laser have been provided by the U.S and the optical components by Germany (Sheard et al. 2012). It allows a much better observation of the change in distance between the two satellites compared to the HAIR microwave observations. First analyses show that the measurement noise is reduced by a factor of about 100 compared to microwave, which is significantly better than the specifications before launch (Abich et al. 2019). The measurement accuracy will thus be in the range of some 10 nm, about half the diameter of a SARS-CoV-2 virus—and this over a distance roughly equivalent to the distance between Potsdam and Hannover.

**Fig. 16:**
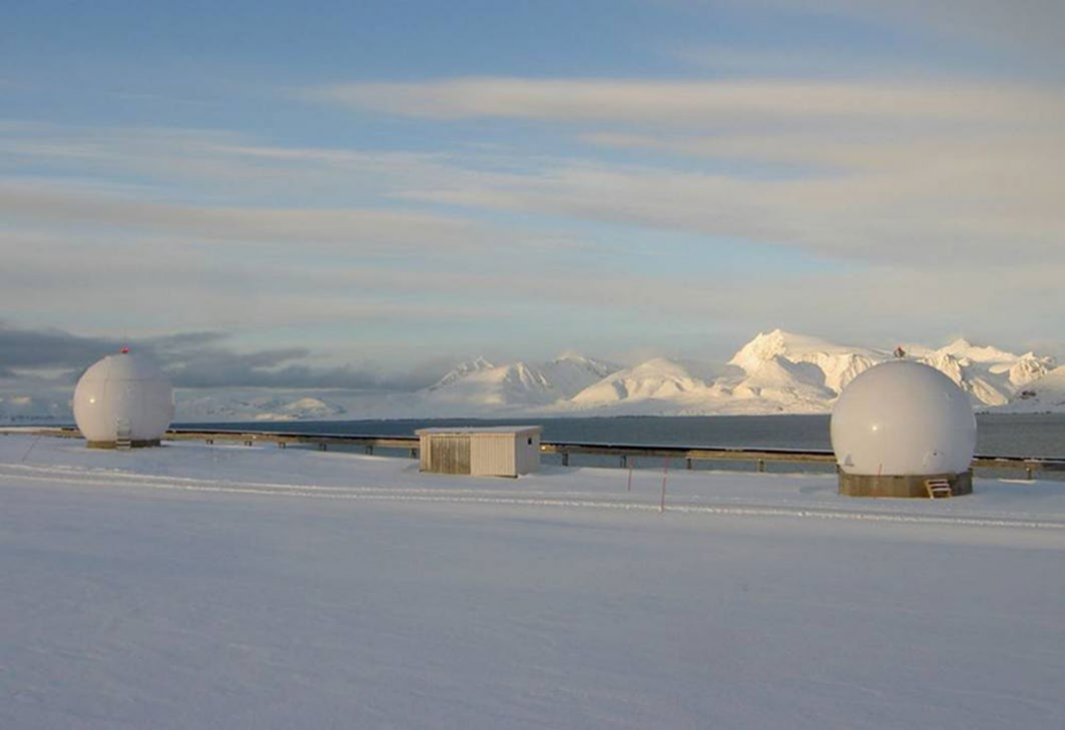
The satellite receiving station Ny-Ålesund with its two antennas (Photo: C. Falck, GFZ)

The mission operation of GRACE-FO will be financed for the first five years by the GFZ and realized by DLR/GSOC as in the case of GRACE. New is that the satellite receiving station of the GFZ in Ny-Ålesund on Spitsbergen (Fig. 16) will be used as primary receiving station. This has the advantage that during each orbit of the satellites there is contact to the ground and therefore the status of the satellites can be controlled much more often than in the case of GRACE. In contrast, the German stations Weilheim and Neustrelitz, which are routinely used by DLR/GSOC, have contact to GRACE-FO only about every eleven hours, which could be a disadvantage in emergency situations.

The nominal lifetime of GRACE-FO will be five years as for GRACE. The current fuel consumption, thruster activities and solar radiation evolution as well as the very good experiences with GRACE let the scientists of course already dream of some additional years of available mass transport data. Nevertheless, it has to be stressed that GRACE-FO has two single points of failure which might also end the mission at any time. The first is the IPU on GRACE-FO-2 which failed shortly after launch, the second is the accelerometer on GRACE-FO-1 which is needed to model non-gravitational forces as the accelerometer on GRACE-FO-2, but which does not meet its specifications (Landerer et al. 2020). The most important characteristics of GRACE-FO are summarized in Table 9.

**Table 9:** Characteristics of GRACE-FO

GRACE-FO	Gravity Recovery and Climate Experiment-Follow-on
Main instrument for gravity field recovery	Low Earth orbiting identical satellite pair with K-Band inter-satellite ranging system HAIRS, low–low SST
Other instruments Technology demonstrator Separation distance of satellite pair Orbit determination Orbit control Orientation in space Measurement of non-gravitational accelerations	Spaceborne geodetic GPS receiver Laser Ranging Interferometer 220 ± 50 km Spaceborne geodetic GPS receiver Laser retro reflector Three stellar sensor systems Electrostatic Super-STAR accelerometer
Mission duration	22.5.2018–
Orbit height	Descending from an altitude of 490 km after launch
Orbit inclination	89° (quasi-polar)
Orbit eccentricity	Quasi-circular

## Future Planning

A disadvantage of the current GRACE concept is that only one satellite pair is flown in a nearly polar orbit with an 89° inclination. Thus, GRACE and now GRACE-FO observe the variations of the gravity field signal essentially only in flight direction on orbits from the North to the South Pole. This non-uniform mapping of the Earth's gravity field (anisotropy) results in stripes in the derived gravity field maps. These disturbances can be corrected by post-processing, but at the same time additional errors are generated and the signal is attenuated. Therefore, the GRACE concept makes it difficult to significantly improve the spatial and temporal resolution despite the successful operation of the LRI on GRACE-FO (Flechtner et al. 2016).

A significant improvement will only be possible by so-called Next Generation Gravity Missions (NGGM). The current NASA Earth Science Decadal Survey Report (National Academies of Sciences 2018) has listed mass transport observations as one of the five main priorities of Earth observation for the next decade. To this end, studies are currently being carried out at NASA, but also at ESA, CNES and DLR/GFZ, to determine which mission concept would best improve the spatial and temporal resolution of gravity field maps but also to secure continuity of data. In order to overcome the anisotropy, it is proposed, for example, to launch a second pair of satellites in addition to the one flying over the polar regions with an inclination of only about 70°. Both pairs of satellites could be equipped with a LRI successfully tested on GRACE-FO. This so-called Bender constellation (after Peter Bender, who first investigated this constellation (Bender et al. 2008)) has been investigated in various simulation studies (e.g. Bender et. al. 2008; Wiese et al. 2009, 2011; Elsaka et al. 2014). It could be shown that the combination of the measurement data of these two satellite pairs using a much lower orbit of about 320 km could improve the accuracy of the gravity field models by a factor of 10 and at the same time increase the spatial resolution from 400 to 150 km. Of particular importance in this context is the improved observation of the non-gravitational perturbation accelerations acting on the satellite, which are not caused by the gravity field but mainly by the air drag-drag and the radiation pressure of the Sun. For GRACE and GRACE-FO these are directly measured with a high-precision accelerometer. However, the accuracy of this accelerometer would have to be further improved. Finally, the models for the correction of tidal and non-tidal short-term (hours to days) mass variations in the atmosphere and in the oceans must be further improved. The GFZ has made a significant contribution to this since the beginning of the GRACE mission (Dobslaw et al. 2017).
